# Discovery of ferroptosis-inducing R4VP compounds for targeting aggressive cancers

**DOI:** 10.1038/s41388-026-03829-2

**Published:** 2026-06-05

**Authors:** Avital Oknin-Vaisman, Deepanjan Panda, Rostislav Novak, Ghazal Kheshaiboun, Eliya Bitman-Lotan, Satish Gandhesiri, Rubina Kazi, Nikolett Pahor, Viktoria von Heyl zu Herrnsheim, Yamen Abu Ahmad, Guy Kamnesky, Thorsten Mosler, Markus E. Diefenbacher, Ashraf Brik, Amir Orian

**Affiliations:** 1 Rappaport Research Institute and Faculty of Medicine, Ruth and Bruce Cancer Research Center (RTICC), Technion-Israel Institute of Technology IIT, Haifa, Israel; 2 Schulich Faculty of Chemistry, Technion-Israel Institute of Technology IIT,, Haifa, Israel; 3 Division of Orthopedic, Rambam Health Campus Center, Haifa, Israel; 4 Institute of Biochemistry II, University Hospital Building, Frankfurt, Germany; 5 Helmholtz Center, Institute of Lung Health and Immunity & German Center for Lung Research, DZL, Munich, Germany; 6 Ludwig Maximilian University and DKTK Munich, Munich, Germany

**Keywords:** Sarcoma, Cell death

## Abstract

Aggressive and therapy-resistant cancers present a significant challenge to treatment and are associated with poor patients’ survival. Identifying molecular pathways and compounds that target these pathways is critical for improving patient outcomes. RNF4, an E3 Ubiquitin ligase, is pivotal for tumorigenesis in part by stabilizing oncoproteins and its role in DNA repair, thereby enhancing cancer cell survival and driving tumorigenesis. Elevated RNF4 levels are associated with poor prognosis in patients with carcinomas, melanoma, and sarcoma. Here, we describe the design and development of R4VPs, dual degrader compounds connecting two E3 ubiquitin ligases; Von Hippel-Lindau protein (VHL) with RNF4. R4VPs promote RNF4 degradation and thereby reduce the levels of its stabilized phosphorylated oncoproteins, while concomitantly eliminating VHL. R4VPs selectively induce ferroptotic cell death in cancer cells, sparing non-tumorigenic and primary cells in part by binding and modifying the anti-ferroptotic selanoproteins GPX4. R4VPs-induced ferroptosis preferentially targeting cells harboring tumor-driving mutations in the EGFR pathway, whereas it does not affect PI3K-transformed cells. As a consequence, R4VPs effectively induce cell death in Receptor Tyrosine Kinase inhibitor-resistant melanoma and primary patient sarcoma cells. Our findings highlight the potential of selective ferroptosis inducers, such as R4VPs, as a therapeutic strategy for hard-to-treat cancers.

## Introduction

Despite significant advances in cancer therapies, the patient’s response to treatment for aggressive, advanced, and/or therapy-resistant tumors remains a challenge. Cancer development and progression are intimately linked to increased oncoprotein stabilization. Moreover, the development of resistance to chemotherapy and molecular treatments such as receptor tyrosine kinase inhibitors (RTKi), and/or immune check-point inhibitors (ICI) in melanoma is associated with increased oncoprotein stabilization [1–8]. These hallmarks are observed in most patients; therefore, overcoming therapy resistance is an unmet clinical need. Thus, the development of tailored precision therapeutics targeting aggressive and therapy-resistant tumors is required for improving patient outcomes.

**Fig. 1:**
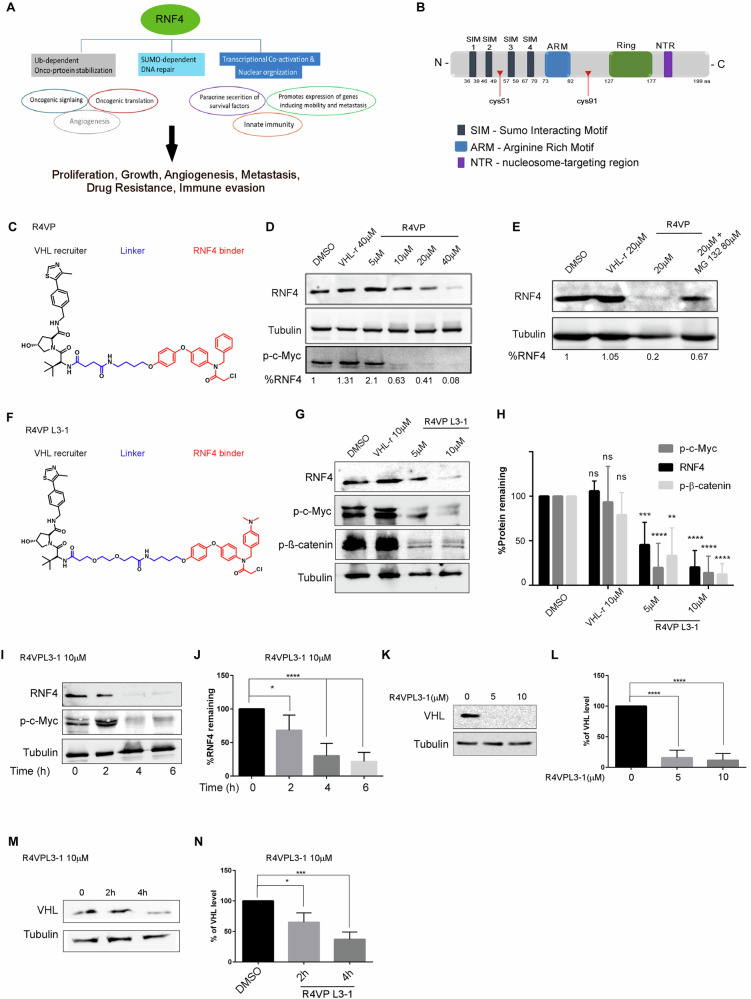
R4VPs force the proteasomal degradation of RNF4 and its stabilized proteins and selectively reduce cancer cell survival. **A** Cancer-promoting activities of RNF4. **B** Schematic diagram of RNF4 structure, red triangles mark amino acid residues Cys51 and 91 that are required for R4VPs binding to RNF4. **C** Chemical structure of R4VP. **D** Western-blot analysis of endogenous RNF4 protein level in extracts derived from B16F10 mouse melanoma cells. VHL-only compound (VHL-r), or R4VP, was added for three hours, and where indicated, 20 μM MG132, a proteasome inhibitor, was added two hours before treatment with the compounds. Tubulin serves as a loading control. **E** R4VP treatment results in a dose-dependent reduction in RNF4 and p-c-Myc levels in B16F10 mouse melanoma cells. **F** Structure of R4VPL3-1. **G**–**J** Western-blot analysis of endogenous RNF4, p^Ser62^-c-Myc, and p^Ser45^-β-catenin proteins levels in extracts derived from A375R PLX4032-resistant human melanoma cells treated with VHL-r or R4VPL3-1 at the indicated concentrations (**G**, **H**), and time (**I**, **J**). **K**–**M** Western-blot analysis of endogenous VHL protein levels in extracts derived from A375R PLX4032-resistant human melanoma cells treated with VHL-r or R4VPL3-1 at the indicated concentrations (**K**, **L**), and time (**M**, **N**). In all experiments, Tubulin serves as a loading control and ****p < 0.0001, ***p < 0.001, **p < 0.01. ns non-significance. RNF4 (*n* = 5), p-c-Myc (*n* = 4), p-b-catenin (*n* = 3). Statistical analysis: **H** 2-way Anova Dunnett’s multiple comparisons test. **J**, **K**, **L**, **N** 1-way Anova Dunnett’s multiple comparisons test. *n* = 3 ****p < 0.0001; ***p < 0.001; *p < 0.1.

We recently discovered a novel ubiquitin-dependent pathway that stabilizes and potentiates oncoprotein activity, promoting tumorigenesis. In this pathway, oncoprotein stabilization requires their phosphorylation by mitogenic kinases regardless of the ubiquitin machinery or the degrons that mediate the degradation of these otherwise short-lived oncoproteins [7, 9–11]. The central enzyme in this pathway is the ubiquitin ligase RNF4, which ubiquitinates these oncoproteins, catalyzing the generation of heterotypic K11- and K33-linked polyubiquitin chains. This atypical ubiquitination results in the stabilization and enhancement of the transcriptional activity of multiple oncogenic transcription factors, such as phosphorylated c-Myc, β-catenin c-Jun, among others [9]. In addition to protein stabilization, RNF4 plays various roles in cancer development. Among these functions are DNA damage repair, nuclear protein control, enhancement of oncogenic translation, and tumorigenic impact on the tumor microenvironment, including fostering angiogenesis (Fig. 1A, B; [10, 12–20]).

RNF4 belongs to a small group of evolutionarily conserved RING ubiquitin E3 ligases, termed SUMO-targeted ubiquitin ligases (STUbL) that ubiquitinate SUMOylated proteins [21–23]. RNF4 has a tumor-suppressive function in the case of acute pre-myelocytic leukemia (APL), partly via SUMO-dependent ubiquitination and degradation of the APL oncoprotein driver PML-RARα [24, 25]. However, in a large group of tumors comprising carcinomas (breast and colo-rectal carcinomas, cholangiocarcinoma, and hepatocellular carcinoma), melanoma, sarcomas, and Myc-driven lymphomas, RNF4 has a pro-tumorigenic role [9–11, 26–28]. We previously characterized the molecular and biochemical actions of RNF4 in vitro, in cellulo, and in vivo. We found that RNF4 enhanced the tumorigenic properties of cancer cells and was essential for cancer cell survival. In melanoma, RNF4 confers resistance to molecular therapy in vitro and in vivo [10]. In sarcomas and melanomas, RNF4 drives the expression of the survival factors BMP6 and its co-receptor RGMb, which are secreted from the tumor cells and act locally, and VEGF that promotes tumor angiogenesis [10, 11]. High levels of RNF4 are observed in about ~30–40% of colon cancer, melanoma and sarcoma biopsies, and are associated with poor prognosis in breast cancer, melanoma, and multiple types of sarcomas. While RNF4 is non-oncogenic on its own, cancer cells are “addicted” to RNF4, and eliminating RNF4 genetically resulted in the death of aggressive and therapy-resistant cancer cells and tumors [9–11]. Thus, RNF4 is potentially an “Achilles’ heel” of multiple tumor entities, which makes it an excellent target for precise cancer therapy.

Inhibition of the enzymatic activity of RING proteins is challenging. Taking a different approach, we decided to eliminate RNF4 via targeted protein degradation (TDP) rather than inhibit its activity by designing specific proteolysis-targeting chimeras (PROTACs; [29–35]). PROTACs are double-headed hetero-bifunctional molecules; on the one hand, they bind to the protein of interest (POI), and on the other hand, they bind to an E3-ligase that catalyzes their ubiquitination. The two modules are connected via a short linker, and proximity results in ubiquitination and proteasomal degradation of the POI. Several E3 recruiters have been discovered and are in advanced clinical trials [34]. Among these E3-recruiters are well-known Cerbelon (Crbn) and the Von-Hipple Lindau (VHL) ubiquitin ligase proteins [30, 36–38]. In addition, RNF4 was suggested to be an E3 recruiter that, when linked via a linker to the small molecule JQ1, induced the degradation of the oncogenic BRD4 transcriptional co-activator and the Hippo pathway regulator YAP [39, 40]. However, compound-driven elimination of RNF4 and their impact on cancer cells has not been reported.

Here, we report the development of RNF4-VHL-Protac-like (R4VPs) compounds that promote the degradation of RNF4, reduce the protein levels of its stabilized phospho-oncoproteins, and, concomitantly, reduce VHL levels. R4VPs induce selective ferroptotic cell death, leading to the rapid death of human melanoma cells resistant to RTKi and sarcoma cells, including patient-derived primary tumor cells, but do not affect non-tumorigenic cell lines or primary Mouse Embryonic Fibroblasts (MEFs).

## Materials and methods

### Chemical synthesis and compound validation

All chemical materials, procedures, chemical structures, and NMR validation are described in detail under the chemical supporting SI.

### Antibodies

α-RNF4 antibody (1:200, sc-517643) and mouse α-tubulin (1:2000, SC-5286) were obtained from Santa Cruz Biotechnology. α-HA antibody was a kind gift from Ami Aronheim. p-c-Myc (1:500, #94015), p-β-catenin (1:300, #9564) and ferroptosis antibody sampler Kit (#29650) were obtained from Cell Signaling Technology (CST). α-4HNE was from Abcam (1:200, #ab46545). Lamin A/C primary labeled antibody (# sc-376248) was from Santa Cruz Biotechnology. Cleaved PARP (Asp214) (D64E10) XP® Rabbit mAb (#5625). Anti-biotin (D5A7) Rabbit Monoclonal Antibody #5597 from CST. Monoclonal Anti-PARP antibody produced in mouse α-P248 and Anti-FLAG® M2 Magnetic Beads - M8823 from Sigma-Aldrich α-GPX4 antibody #52455 was from CST.

### Biological materials and compounds

RNF4 purified from bacteria was purchased from Boston Biochem (#E3-210), and Image-iT™ Lipid Peroxidation Kit from Thermo Fisher Scientific (#C10445). FITC-Annexin V (#BLG-640906) and Annexin V binding buffer (#422201) were purchased from ENCO. Propidium iodide (PI) was purchased from Sigma-Aldrich (#P4170-10MG); 5-TMRIA [Tetramethylrhodamine-5-iodoacetamide] was from Megapharm (#AS-81410). BRAF inhibitor PLX4032^®^ was purchased from Selleckchem (#S1267). Ubiquitin E1 inhibitor (TAK-243), Deferasirox #HY-17359 and ALDH3A1-IN-3 #HY-125085 were from MedChem Express. MG132, a proteasome inhibitor (#MBS474790), was purchased from Mercury. Phosphatase inhibitor (#4906845001) and protease inhibitor (#11873580001) cocktails were from Sigma-Aldrich. EZ-view red αHA-gel beads (#E6779) were from Sigma-Aldrich. The live cell imaging solution (#A14291DJ) was from Invitrogen. C11-BODIPY 581/591 fluorescence ferroptosis sensor (#27086) was from Cayman Chem. RSL3 was purchased from TAMAR Laboratory supplies. Necrostatin-1s #17802 was purchased from Cell Signaling.

Materials used for RNA-seq: NEBNext Ultra II Directional RNA Library Prep Kit for Illumina #E7760 and mRNA Magnetic Isolation Module (#E7490) were from NEB and RNA kit (# 5067- 5576) of Agilent. dsDNA HS Assay Kit (#Q32854) was purchased from Invitrogen.

### Plasmids used in this study

pcDNA3 HA-hRNF4 was as in ref. [9]. pcDNA3 RNF4^C51A^, pcDNA3 RNF4^C91A^, RNF4^C51,91A^ were generated by quick-change site-directed mutagenesis^®^, according to the kit guidelines, using the relevant primers and validated by sequencing. GST-RNF4 and GST-RNF4^DM^ were generated by a similar approach using pGEX6-RNF4. All constructs were validated by sequencing. EGFR^L858R^ (addgene number: #11012, pBabe puro HA-PIK3CA^H1047R^ (addgene number: #12524), pBabe puro HA-PIK3CA^E545K^ (addgene number: #12525) and pBabe puro BRAF ^V600E^ (addgene number: #15269).

**Primers used in this study are listed below:**

RNF4^C51>A^ Fwd.5’-gagatgaaattgtggacctcactgctgaatctttagagcctgtg-3’

RNF4^C51>A^ Rev.5’-gacaggctctaaagattcagcagtgaggtccacaatttcatctc-3’

RNF4^C91>A^ Fwd.5’-ggaccatgctgacagcgctgtggtgagcagtgac-3’

RNF4^C91>A^ Rev. 5’-gtcactgctcaccacagcgctgtcagcatggtcc-3’

**sgRNA design for targeting RNF4, VHL, or both**:

sgRNAs were designed using the CRISPRtool (https://zlab.bio/guide-design-resources).

RNF4 Fwd:

5’-tatcttGTGGAAAGGACGAAACACCgacgctttctctgagtagcaGTTTTAGAGCTAGAAATAGCAAGT-3’

RNF4 Rev:

5’-ACTTGCTATTTCTAGCTCTAAAACctcaccgtcaacaatcttgtcGGTGTTTCGTCCTTTCCACaa-3’

VHL Fwd:

5’TATCTTGTGGAAAGGACGAAACACCGCCCGTATGGCTCAACTTCGAGTTTTAGAGCTAGAAATAGCAAGTTA-3’

VHL Rev:

5’AACTTGCTATTTCTAGCTCTAAAACGGCCCGTACCTCGGTAGCTGCGGTGTTTCGTCCTTTCCACAA-3’

### Methods and experimental design

#### Gel-based 5-TAMRA-Iodoacetamide (IA) displacement assay

The binding of bacterially purified RNF4 to the indicated RNF4 binding moieties (R4Bs) was determined using 5-TMRIA displacement assay similar to ref. [39]; 200 ng human RNF4 was incubated with the R4B molecules at the indicated concentrations in PBS solution. Reactions were incubated for 30 min at RT. Subsequently, 250 nM of the 5-TMRIA-Tetramethylrhodamine-5-iodoacetamide,(dissolved in DMSO) was added and incubated for 1 h incubation at RT with a total reaction volume of 40 μl. Binding was terminated by adding 8 μL of 5× Laemmli protein sample buffer and heating at 90 °C for 5 min. The samples were resolved over a 12.5% SDS-PAGE. Fluorescent imaging was performed on LAS4000 (Image Quant) and quantified by ImageJ.

#### Cultured cell line transfections and infections

A375R, HEK293, were as reported in ref. [10]. MEFs were a kind gift of Yuval Shaked, RTiCC. All other cell lines were obtained from ATCC. Beas2B, HaCat, SCC1, and 143B were maintained in DMEM with 100U/ml penicillin, 0.1 mg/ml streptomycin, Glutamine 4 mM, and 10% FBS. A375R were cultured in DMEM with 10% FCS, penicillin/streptomycin, and 2 µM PLX4032, a Vemurafenib^®^ analog. The BEAS2B oncogenic cell line is an early oncogenic transformation stage of lung cancer (BEAS-2B EGFR^L8585^, BEAS-2B^BRAF-V600E^, BEAS-2BPIK3^H1047R^ were as previously described [41]. All cell lines were authenticated by STR profiling. Cells were transfected using CalFectin transfection reagent according to manufacturer instructions (Sinagen Laboratories).

#### Crisper CAS9 gene-editing in BEAS-2B transformed cells

Double sgRNA array cloning: The target vector pAAV sgRNAXhoI-EFS-SpCas9-P2A-Puro (sequence available in this paper S6) was generated using a gBlock (IDT) containing the EFS short promoter, a P2A cleavage site and the Puromycin resistance gene. Between the AAV ITR and the EFS promoter was the standard U6-sgRNA cassette cloned using the pLenti-CRISPR-V2 vector as a template. Using an oligo, we introduced an XhoI restriction site to separate the U6 promoter from the trcRNA. Using the above-mentioned oligos, we next amplify a double sgRNA cassette using the STAGR method (https://journals.plos.org/plosone/article?id=10.1371/journal.pone.0196015) and amplified using 2xPhanta Polymerase (https://absource.de/2-Phanta-Max-Master-Mix/VB-P515). The double sgRNA cassette was seamless cloned into the target vector via the XhoI site using the Exnase MultiS ligation system, according to manufacturer protocol (https://absource.de/ClonExpress-MultiS-One-Step-Cloning-Kit/VB-C113). The inserts were confirmed using Sanger sequencing.

DNA transfection and infection: For DNA transfection, a mix of 2.5 μg plasmid DNA, 200 μl free medium and 10 μl PEI was added into the 6-well dish medium (60% confluence), after 6 h incubation at 37 °C the medium was changed to full supplemented medium. Transfected cells were selected with 5 μg/ml puromycin for 72 h. Resistant cells were propagated and subjected to multiplex immunofluorescent imaging as described shortly: Immunofluorescence (High Content) of BEAS-2B cells : Oncogenic BEAS 2B-derived cell lines were transiently transfected with the knock-out constructs VHL-Puro and RNF4-Puro as well as the vector control. Transfected cells were seeded at a confluency of 70% into 96-well plates (Thermo Scientific Nunclon™ Delta Surface) and cultivated at 37 °C, 5% CO_2_ and 95% humidity. Afterwards, cells were washed with PBS and fixed with PFA (4% in PBS) for 7 min at room temperature. The samples were permeabilized with PBS 0.4% Triton X-100 for 4 min, washed with PBS and blocked for 1 h at room temperature with 5% BSA in PBS. To stain, antibodies (RNF4: 17810-1-AP, Proteintech, lot: 00056102 ; GPX4: 67763-1-IG, Proteintech, lot: 10027815; VHL: 24756-1-AP, Proteintech, lot: 00021179) were diluted 1:100 in 1% BSA in PBS. The samples were stained overnight at 4 °C with the antibodies, respectively. Cells were washed with PBS and incubated with the secondary antibody (1:500 in 1% BSA in PBS) and DAPI (1:1000 in 1% BSA in PBS) for one hour. Subsequently, the staining was imaged using a high content screening platform and analyzed with HCS Navigator ™ (ThermoFisher; version 6.6.4 (build 8616)). Data was analyzed and visualized in RStudio (version 2023.09.1 build 494 based on the R-version 4.3.1).

#### In vitro bio-R4VPL3-1 binding assays

1 × 10⁶ HEK293 cells were transfected with plasmids coding for the expression of HA, or HA-RNF4, or HA-RNF4^C51A;C91A^ (termed RNF^DM^) using CalFectin according to the manufacturer’s protocol. 48 h post-transfection, cells were harvested and washed three times with ice-cold PBS and lysed in mild lysis buffer (10% glycerol, 1% Triton X-100, 150 mM NaCl, 1 mM EDTA, 20 mM HEPES pH 7.4 supplemented with protease inhibitors). Lysates were sonicated once and centrifuged at 13,000 RPM for 15 min at 4 °C. The supernatant was collected and transferred to a fresh tube. For each of the samples, 1 mg of total protein extract was incubated with α-HA beads in TPA buffer (30 mM tris pH 7.5, 100 mM NaCl, 5 mM MgCl_2_, 2 mM DTT, 10% glycerol, 0.01% IPGAL and supplemented with protease inhibitors) for two hours at 4 °C with rotation. At this stage, Biotin-R4VPL3-1 was added to a final concentration of 3 µM in a total volume of 500 µl, and binding was performed for 1 h at 4 °C. Subsequently, the beads were washed three times with TPA buffer. Finally, the beads were resuspended in 30 µl of protein sample buffer and resolved by SDS-PAGE. Biotinylated-RNF4 was detected via western-blotting using α-biotin and α-HA (for 10% input samples).

In vitro Biotin-R4VPL3-1 interaction with RNF4 was performed using recombinant GST-RNF4 and GST-RNF4^DM^ proteins: GST-RNF4 and GST-RNF4^DM^ were generated similarly to what was described in ref. [9]. GST, GST-RNF4, or GST-RNF4^DM^ were incubated together with Biotin-R4VPL3-1 in TPA buffer with protease inhibitors in a 50 μl reaction incubated for 30 min in RT. Subsequently, 10 μl strep-avidin beads were added, and the volume was adjusted to 500 μl and incubated in 4 °C overnight. Samples were centrifuged and washed three times with 1 ml of TPA buffer supplemented with protease inhibitors. 30 μl of sample buffer was added and heated at 95 °C for 5 min. Samples were resolved over SDS-PAGE, and biotinylated proteins were detected using western blot analysis and α-biotin antibody.

Binding assay of R4VPL3-1 or R4VPL3-1^chloro^ to GPX4 was performed using cells overexpressing FLAG-VHL treated with either 3 μM R4VPL3-1 or R4VPL3-1^Chloro^ (that lacks the reactive chloro atom within the RNF4 binder moiety). Cells were lyzed using lysis buffer (10% glycerol 1% triton, 150 mM NaCl, 10 mM EDTA 0.5 M, 20 mM Hepes 1M pH-7.4), followed by immunoprecipitation using FLAG-Sepharose beads and western blots analysis using αGPX4 antibody.

#### Cell viability

Indirect cell viability was determined using CellTiter-Glo® Luminescent Cell Viability Assay (ATP-Lite; Promega). In brief, 10^3^ cells were seeded in triplicates for each time point in white 96-well plates with transparent bottoms and cultured for the indicated time. 30 μL CellTiter-Glo® solution was added to each well, and viability was quantified by monitoring luminesce using a Plate Reader according to the kit protocol.

#### Sphere formation assay

A375R, SCC1, 143B cancer cells, MEF, and HaCaT cells (10^3^ cells/well) were seeded in 6-well plates in 2 ml of the appropriate growth media and maintained at 37 °C in a humidified incubator. After eight days, cells were gently washed once with PBS and fixed for 1 h with 5% formaldehyde, washed trice with PBS, stained with 0.05% crystal violet for 20 min, photographed, and counted.

#### Live cell proliferation

2 × 10^3^ BEAS2B transformed cells expressing the indicated oncogenic driver were seeded in 96-well plates. The following day, cells were treated with R4VPL3-1 or DMSO (solvent control) and were incubated for 72 h. Subsequently, cells were fixed using ice-cold 100% Methanol (Sigma-Aldrich) for 10 min on ice, washed three times with PBS for 5 min each, and blocked with 5% BSA in PBS, for 1 h at RT. Afterward, cells were incubated with α Lamin A/C primary GFP-labeled antibody and kept in the dark for 1 h at RT, followed by three washes with PBS. Imaging was carried out using the LI-COR Odyssey M Imaging System, which detects the intracellular fluorescent signaling in whole cells.

### Lipid peroxidation measurements

BODIPY™ 581/591 C11-fluorescence ferroptosis sensor was used to determine live lipid peroxidation in A375R cells according to the manufacturer’s recommendations. In brief, 5 × 10⁴ A375R cells were plated in High-Content Imaging Glass Bottom 24-well Microplates for ∼24 h and then treated with either 3 µM R4VPL3-1 for 2.5 h or 100 µM Cumene hydroperoxide for 2 h (positive control, an established lipid-peroxidation inducer). Where indicated 10μM Ferrostatin 1 (Ferr-1) were added to the culture media 1 h prior to the addition of R4VL3-1. Subsequently, 10 μM lipid peroxidation sensor BODIPY™ (581/591)C11 was added for 30 min together with 1 mM Hoechst 33342 in DMEM full media. Cells were gently washed twice with PBS and incubated in live cell imaging solution (Invitrogen). Fluorescence was measured at 581/590 nm (excitation/emission) for the reduced dye (red), and at 488/510 nm (excitation/emission) for the oxidized dye (green), while Hoechst was measured at 350/461 nm (excitation/emission) using confocal imaging. The ratio of green-to-red fluorescence intensity was calculated to determine the extent of lipid peroxidation. The values were normalized to the number of cells in each well using Hoechst staining. Confocal microscopy images of live cells stained with BODIPY™ (581/591)C11were acquired using the LSM880 (Zeiss), and quantification was performed the ZEN Imaging Software (Zeiss).

Determination of lipid peroxidation using α-4HNE antibody. 10^5^ A375R cells were grown on coverslips in a 24-well plate and were treated with 3 μM R4VPL3-1 as indicated for two hours. Where indicated, 10 μM Ferrostatin 1 (Ferr-1) were added two hours before RV4Ps treatment. Cells were washed with DMEM and fixed with 4% paraformaldehyde (PFA) for 15 min at room temperature on a rotator. After fixation, the PFA was aspirated, and the cells were washed three times with PBS. Blocking was performed using a solution of 10% normal goat serum (NGS) and 0.3% Triton X-100 in 1× PBS for two hours at room temperature on a shaker to reduce nonspecific antibody binding. Primary antibody α-4HNE, a marker of lipid peroxidation, was diluted in a solution containing 5% NGS and 0.1% Triton X-100 in 1× PBS at a final concentration of 1:200. Cells were incubated with the primary antibody solution overnight at 4 °C on a shaker. The following day, coverslips were washed five times with PBS for 10 min each on a shaker. Secondary α-Rabbit antibody was diluted 1:1000 in PBS, including DAPI to mark nuclei. Coverslips were incubated with the secondary antibody for 1 h at room temperature on a rotator. Post-incubation, the coverslips were washed three times with PBS for 10 min. Cells were mounted with a mounting medium, and coverslips were placed on slides. Fluorescence images were acquired using a Zeiss LSM700 confocal microscope.

#### Preparation of protein extracts

Cells were washed three times with ice-cold PBS and harvested in RIPA buffer supplemented with 10 mM EDTA pH8.0 along with protease and phosphatase inhibitors (Sigma-Aldrich). Cell extracts were sonicated using a microtip for 10” followed by centrifugation for 20 min at 13,000 RPM at 4 °C. Extracts were resolved over SDS-PAGE, and proteins were identified using the indicated antibodies and visualized and quantified using chemiluminescence LAS4000 and Image-Quant software. Fold changes in protein levels were calculated relative to Tubulin.

#### Cell death analysis using flow cytometry

Cell death was determined by FACS using Annexin V-fluorescein isothiocyanate (FITC)/PI using a flow cytometer. A375R Cells were trypsinized, centrifuged, and washed with cold PBS. Subsequently, cells were resuspended in 500 μL Annexin binding buffer, mixed with 5 μL Annexin V + FITC and 20 μL PI, followed by incubation at RT for 15 min in dark. Cells were analyzed using a flow cytometer following the manufacturer’s protocol. Annexin V and/or Propidium Iodide-labeled cell population were counted by flow cytometer.

#### Establishment of patients-derived sarcoma cells

Patient-derived sarcoma cells were derived from tumors during onco-orthopedic resections of non-treated patients, and according to Helsinki committee permit # RBM-0536-22 and signed patient informed consent. Tumor specimens were collected in a 50 mL sterile tube with 20 mL DMEM medium. Samples were washed with PBSx2 and minced into the smallest possible fragments. In parallel, part of the surgical specimens was analyzed by an experienced sarcoma pathologist, and the exact nature of the tumor was reconfirmed. Tumor slices were seeded on a 10 cm dish, and re-sliced, and seeded in 10 cm plate. Attached cells were maintained in DMEM supplemented with 20% FBS, 1% glutamine, and 1% Penicillin/Streptomycin, and were passaged once at 85–90% confluence by washing with PBS, then seeded for experimentation at a split ratio not exceeding 1:3. 10^3^ cells were used for proliferation and SFA assays.

### RNA-seq and data analyses

RNA-Seq was performed in a set of three conditions using A375R cells and three independent biological repeats; We compared differentially expressed genes (DEGs) between DMSO-treated A375R cells (control), to cells treated with 10 µM R4VPL3-1 (the full PROTAC-like molecule) or with 10 µM VHL-r (the VHL binding moiety alone) for 2 h. RNA extraction was performed using MACHEREY-NAGEL NucleoSpin RNA, Mini kit for RNA purification. RNA quality was evaluated using the TapeStation 4200 (Agilent) with the RNA kit. The RINe values of all samples were in the range of 9.3–10, indicating high quality. According to the manufacturer’s protocol, libraries were constructed simultaneously using the NEBNext Ultra II Directional RNA Library Prep Kit for Illumina. 800 ng total RNA was used as the starting material. mRNA pull-down was performed using the NEBNext® Poly(A) mRNA Magnetic Isolation Module. RNA-seq library QC was performed by measuring library concentration using Qubit (Invitrogen), with the dsDNA HS Assay Kit (Invitrogen) and size determination using the TapeStation 4200 with the High Sensitivity D1000 kit. All libraries were then mixed into a single tube with equal molarity. The RNA-seq data was generated on Illumina NextSeq2000, using P2 100 cycles (Read1-100; Index1-8; Index2-8) (Illumina, #20046811). Single reads (100 bps) were aligned to the Homo sapiens (GRCh38.109) reference genome using STAR (V2.5.3a) with a mismatch ratio allowed <0.2; the minimum and maximum intron sizes were set to 20 and 1,000,000, respectively. The number of reads per gene was counted using Htseq-count (v2.0.2) with ‘reverse’ mode. Normalization and differential expression analyses were conducted using DESeq2 R package (v1.36.0). The similarity between samples was evaluated within DESeq2 package using a Euclidean distance matrix (shown in a heatmap with a clustering dendrogram) and a principal component analysis (PCA). The latter is drawn from the most variable genes (50, 500, 1000, 2000, 5000, 10,000 genes). Two factors determine the threshold for significantly differentially expressed genes: FDR-adjusted *p*-value ≤ 0.05 (for mouse B16 experiment, a *p*-value ≤ 0.01 threshold was specifically chosen since the DE analysis yielded over 4000 differential genes) and the ‘base-mean independent filtering’ threshold, which is calculated by the DESeq2 algorithm for each comparison. FDR was calculated using the default approach of DESeq2, Benjamini–Hochberg (BH). Since in both experiments, no major batch effect was indicated, a simple single-factor model was applied, and lists of DEGs were created for all the possible contrasts.

#### Data analysis and bioinformatics tools

For RNA-seq analysis, Gene ontology analyses were performed using Cytoscape software, including the ClueGO app (v2.5.10) in Cytoscape (v 3.10.1) was used to conduct GO enrichment analyses. We used ClueGO to identify different functional groups in the following terms: 1. Biological Process (BP) A *p*-value ≤ 0.001 was used as the cut-off criterion; 2. KEGG A *p*-value ≤ 0.05 was used as the cut-off criterion. Ingenuity Pathway analysis (IPA) was performed as previously described [10].

#### Statistical analysis

Details are provided in the relevant figures and legend. Protein levels as measured using Western blot analysis were quantified using Image Quant. All experiments were performed at least in three independent biological replicates unless stated otherwise, and representative images are shown.

## Results

### R4VP-mediated proteasomal degradation of RNF4

As a starting point, we tested whether eliminating RNF4 using a degrader molecule would be a suitable strategy to eradicate cancer cells. We used the previously reported RNF4-binding molecule CCW16, termed here L1 [39], and linked it to an established VHL recruiter moiety (VHL-r), thereby creating a PROTAC-like molecule that we termed R4VP (Fig.1C; for details regarding the synthesis of all compounds and their validation see Chemical SI). Next, we evaluated the steady-state protein levels of endogenous RNF4, and it stabilize target oncoprotein c-Myc in murine B16F10 melanoma cells upon treatment with either R4VP or VHL-r alone, respectively. Treatment with R4VP (3 h), but not VHL-r, resulted in a dose-dependent reduction in endogenous RNF4 protein level, that was inhibited by the proteasome inhibitor MG132 (Fig. 1D, E), confirming that R4VPs induces the proteasomal degradation of RNF4 in B16F10 cells.

### Anti-cancer activities of R4VP

To test the anticancer activities of R4VP against cancer and non-tumorigenic cells, we treated a set of cell lines with increasing concentrations of R4VP or VHL-r. We observed that treatment with R4VP, but not VHL-r, inhibited the proliferation and sphere formation (SFA) of A375R, a human melanoma cell line that is resistant to PLX4032, a Vemurafenib^®^ analog, as well as SCC1 human squamous skin cancer cells. Moreover, an inactive R4VP lacking the reactive chloro-atom that mediates the covalent reaction of R4VP with RNF4, showed no anti-proliferative activity (Supplementary Fig. S1A–F). Notably, R4VP did not affect the sphare formation of non-tumorigenic human keratinocytes cell line HaCaT. However, while both types of cells express RNF4 and VHL proteins, the level of RNF4 is lower in HaCaT compared to SCC1 cells, and VHL protein is high in HaCaT compared to SCC1 (Supplementary Fig. S1G, H).

To improve the activity of R4VP, we focused on the RNF4 binding moiety (R4B) that binds to RNF4. We prepared a focused compound-library, based on L1, for in vitro screening and identified L3 as an improved binder of RNF4 (Supplementary Fig. S1B, Chemical SI). Since the structure of the linker is critical for the function of degraders [42], we also modified the original R4VP linker region and generated a series of R4VPs: R4VPL3-1 (Fig. 1F), R4VPL3, R4VPL3-2, and R4VPL3-3 (Supplementary Fig. S2; Chemical SI). While R4VPL3-2 was inactive, R4VPL3-1 and R4VPL3-3 exhibited similar potent anti-proliferative activity without affecting non-tumorigenic cells or MEFs (see below and not shown).

### Induced protein degradation and anti-cancer activity of R4VPL3-1

We characterize the newly developed R4VPL3-1 and tested its impact on the protein level of endogenous RNF4 and the phosphorylated oncoproteins substrates that RNF4 stabilizes, such as p^Ser62^-c-Myc and p^Ser45^-β-catenin [9]. We observed a dose and time-dependent decrease in the protein levels of RNF4 and its stabilized substrates upon treatment with R4VPL3-1(Fig. 1F–J). R4VPs connect two ubiquitin ligases, RNF4 and VHL. Therefore, we also tested whether R4VPL3-1 reduces VHL levels. Indeed, both VHL and RNF4 are stabilized by the proteasome inhibitor MG132, and R4VPL3-1 induced dose- and time-dependent reduction in the abundance of VHL proteins in A375R cells (Fig. 1K–N, Supplementary Fig. S1I).

**Fig. 2:**
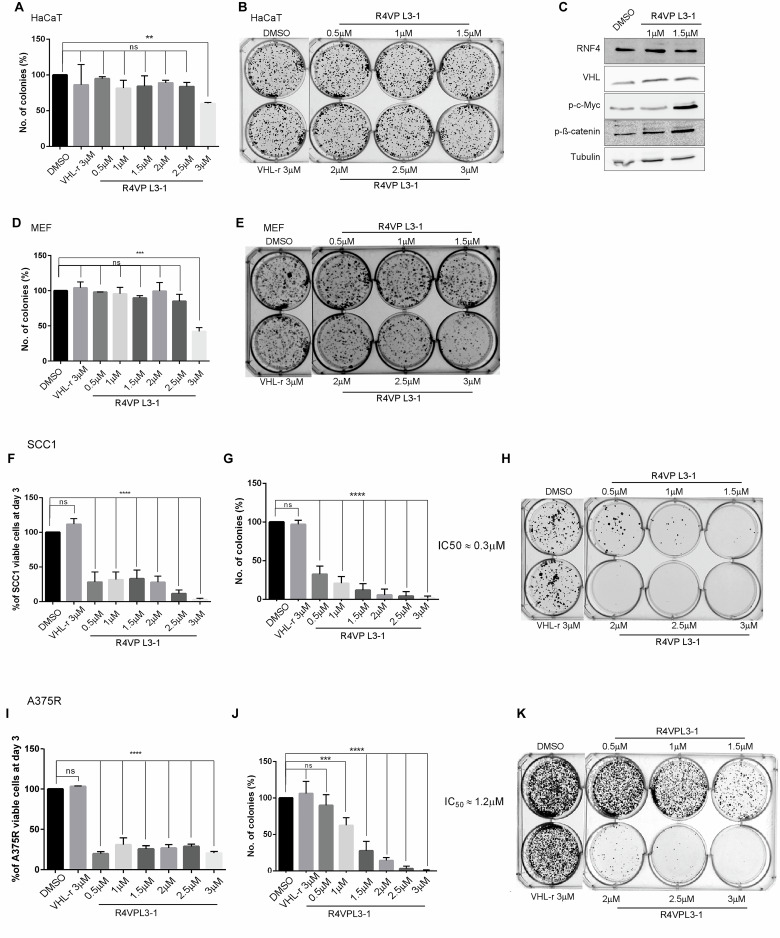
R4VPL3-1 induces death of human skin and melanoma cancer cells, but does not affect MEFs and non-oncogenic cells. **A**, **B** Sphare formation (SFA) of HaCat, a non-tumorigenic skin keratinocyte cell line upon treatment with either VHL or R4VP3-L1 in HaCat (**A**, **B**). **C** Endogenous RNF4 and VHL, p-c-Myc and p-β-catenin protein levels in the above cells. **D**, **E** SFA of mouse embryonic fibroblasts (MEFs) in both cells. SFA is only minimally inhibited by R4VPL3-1. Proliferation (**F**, **I**) and SFA (**G**, **H**, **J**, **K**) of SSC1 human squamous skin carcinoma cells (**F**–**H**) or human PLX4032-resistant A375R human melanoma cells (**I**–**K**) is attenuated upon treatment with R4VPL3-1 at the indicated doses but not upon treatment with DMSO or VHL-r compound.

We tested the biological activity of R4VPL3-1 and examined whether it exhibits selective anticancer activity. We found that R4VPL3-1 had no activity towards non-tumorigenic HaCat keratinocyte cell line, or primary mouse embryonic fibroblasts (MEFs, Fig. 2A–E). We observed that in contrast to A375R melanoma cells in HaCaT cells the levels of RNF4, VHL, and RNF4 stabilized proteins p-c-Myc and p-β-catenin, were not reduced upon treatment with R4VPL3L-1 (Fig. 2C). R4VPL3-1 has significant anti-cancer activity towards human squamous cancer cells (SCC1), inhibiting proliferation and SFA with an IC_50_ of ~0.3 μM (Fig.2 F–H).

### R4VPL3-1 induced rapid cell death of RTKi-resistant melanoma cells

**Fig. 3:**
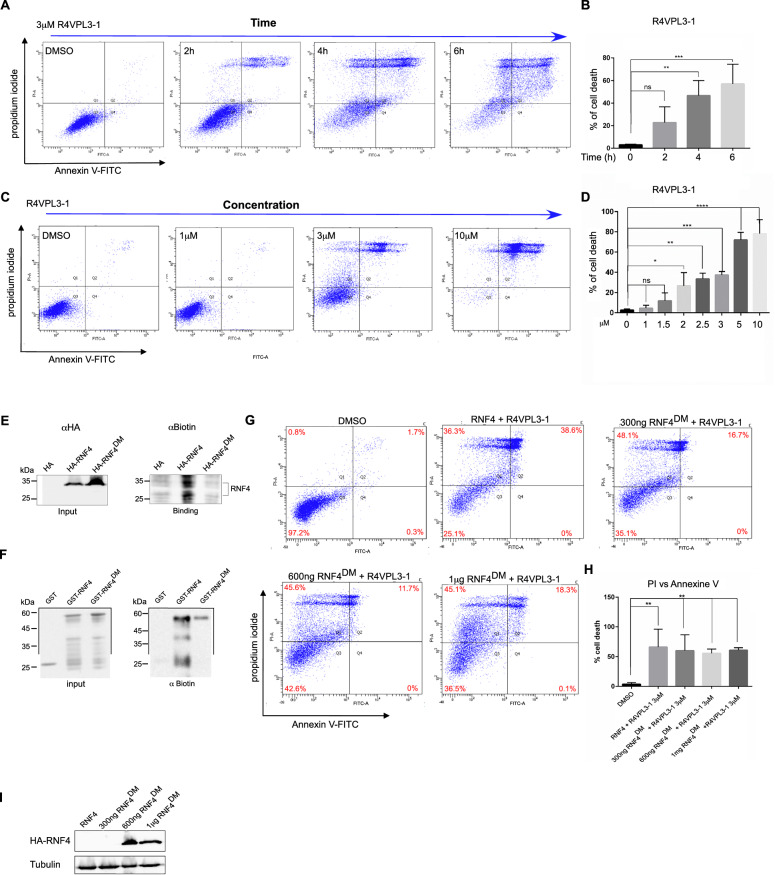
R4VP3L1 induces cell death of RTKi-resistant A375R human melanoma cells. **A**–**D** Cell death was determined using FACS analysis using propidium iodide and Annexin V-FITC. **A**, **B** Time-course of cell death upon treatment with 3 μM R4VPL3-1. **C**, **D** Dose-dependent treatment with R4VPL3-1 for four hours, and **A**, **C** are representative experiments, and the full titration of R4VP3L-1 is shown in (**D**). **E** HA-RNF4^DM^ (RNF4^C51A,C91A^) fails to bind to biotin-R4VPL3-1. HA-RNF4 or RNF4^DM^ was expressed in HEK293 cells and immunoprecipitated using HA-bead. Subsequently, the binding of Biotin-R4VPL3-1 was determined in vitro and analyzed via western blot analysis, using α-Biotin antibody, and 10% input is shown. **F** Western blot analysis of in vitro binding of GST-RNF4, or GST-RNF4^DM,^ to Biotin-R4VPL3-1 using α-Biotin antibody. **G**, **H** Expression of increasing amounts of HA-RNF4^DM^ mutant does not cancel R4VPL3-1-induced cell death. **H** is a quantification of three independent biological experiments. **I** HA-RNF4^DM^ protein levels in a representative experiment performed in (**H**), and Tubulin serves as a loading control. Statistical analysis was performed using one-way Anova Dunnett’s multiple comparisons tests (**A** *n* = 2; **E** *n* = 4; **G** *n* = 3).

One major clinical challenge is overcoming therapy resistance [2–4]. RNF4 was shown to confer resistance to Receptor tyrosine kinase inhibitors (RTKi) melanoma cells, such as human A375R in cellulo and in vivo [10]. Therefore, we tested whether R4VPL3-1 can overcome RTKi resistance of these A375R cells. R4VPL3-1 inhibited the proliferation and SFA of aggressive A375R under PLX4032 treatment (Fig. 2I–K). To gain insights regarding the inhibitory effects of R4VPL3-1 and potential induction of cell death, we performed FACS analysis using Annexin V and Propidium Iodide (PI) staining. Treatment with R4VPL3-1 induced rapid cell death of A375R cells, resulting in >50% cell death in a time and dose-dependent manner (Fig.3A–D).

To validate that R4VPs-induced cell death is due to the loss of RNF4, we first mapped the binding site(s) of R4VPL3-1 to RNF4 using a biotin-tagged R4VPL3-1, in which the VHL-r moiety was replaced with biotin (Biotin-R4VPL3-1). Subsequently, we tested whether this mutant binds to RNF4 and whether its expression prevents cell death induced by R4VPL3-1. Mass spectrometry analyses identified that biotin-R4VPL3-1 binds to Cys51 and Cys91 within RNF4 (Gotthardt & Müller, personal communication). We validated this finding by comparing the ability of RNF4 or the RNF4 double mutant HA-RNF4^C51A, C91A^ (termed HA-RNF4^DM^) to bind Biotin-R4VPL3-1. We overexpressed HA-RNF4 or HA-RNF^DM^ in HEK293 cells and affinity-purified these proteins from cell extracts using α-HA~beads. Subsequently, we performed in vitro binding assay to Bio-R4VPL3-1. As shown in Fig. 3E, while HA-RNF4 binds to Bio-R4VP3-1, HA-RNF4^DM^ did not bind. We also found that Bio-R4VPL3-1 binds well to bacterially expressed and purified GST-RNF4 but only minimally binds to GST-RNF4^DM^ (Fig. 3F). Next, we tested whether the expression of RNF4^DM^ will protect A375R cells from R4VPL3-1-induced cell death. We found that increasing amounts of HA-RNF4^DM^ did not prevent R4VPL3-1-induced cell death (Fig. 3G–I, see discussion).

### R4VPL3-1 induces ferroptosis in cancer cells

**Fig. 4:**
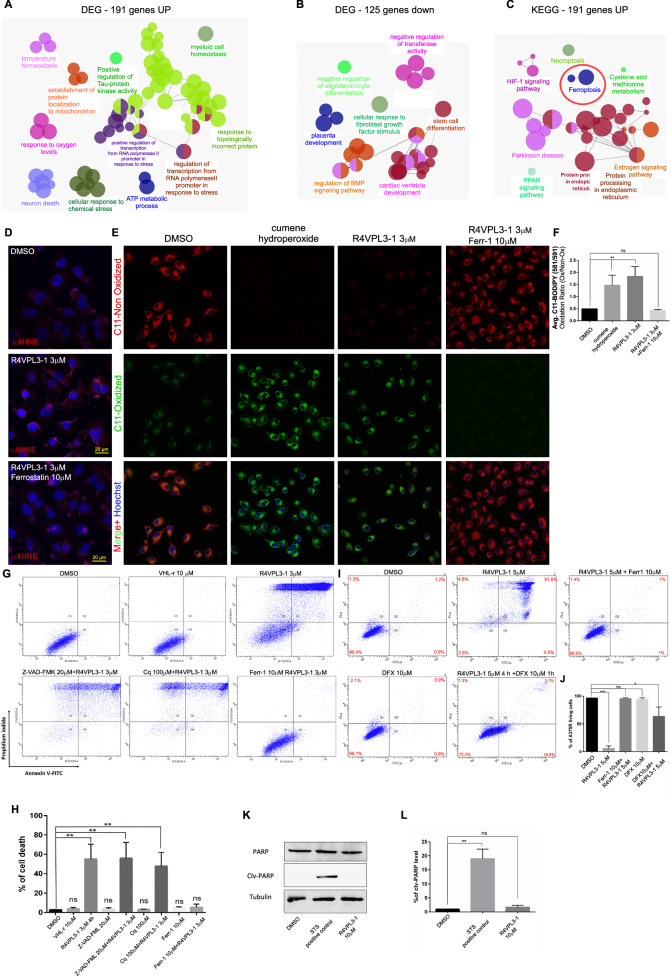
R4VPL3-1 induces ferroptosis of A375R RTKi-resistant melanoma cells. **A**–**C** Cytoscape and KEGG analysis of RNA-seq results identifying statistically significant upregulated and repressed pathways, as well as a cellular process affected by two hours of treatment 10 μM of R4VPL3-1 compared to VHL-r treated A375R cells. In (**C**), the red circle marks ferroptosis, and the complete gene lists are in Supplementary Table S1. **D** 3μM R4VPL3-1 induces lipid peroxidation that is inhibited by 10μM Ferrostatin-1 (Ferr-1), a specific ferroptosis inhibitor, as evident by α-4HNE immune-staining. **E**, **F** Bodipy™-C11 sensor was used to visualize lipid peroxidation induced by R4VP3L-1, which Ferr-1 inhibits. **E** are representative confocal images, the Hoechst stain is blue, and the scale bar is 10 μm. Quantification of shifts in fluorescence measurements are shown in (**F**). Cumene hydroperoxide is a lipid peroxidation compound (positive control); SD are shown of three repeats and ****p < 0.0001. **G**, **H** FACS analysis of R4VPL3-1 induced A375R cell death. 10 μM Ferr-1, or 10 μM DFX, but not DMSO, 20μM Z-VAD-FMK or 100μM Chloroquine (Cq), inhibited R4VPL3-1 induced cell death. **G**, **I** are representative experiments, and quantifications are shown in (**J**, **H**). Statistical analysis: *n* = 3 and ****p < 0.0001, ***p < 0.001, **p < 0.0, ns no-significance. One-way Anova Dunnett’s multiple comparisons tests (*n* = 2). **K**, **L** R4VP3L-1 does not induces cleavage of PARP. Western blot analysis of protein extracts derived from A374R cells treated with either Sterosproine (STS, positive control) or R4VPL3-1 and cleaved-PARP, an indicator of apoptotic cell death, was determined using PARP and cleaved-PARP antibodies. **K** is a representative experiment, and quantification is shown in (**L**).

To identify the cellular pathways involved in R4VPL3-1-induced cell death, we performed transcriptional analysis using whole transcriptomic sequencing and compared A375R cells treated for two hours with 10 μM VHL-r or 10 μM R4VPL3-1 (Fig. 4A–C, Supplementary Fig. S4). We identified 316 differentially expressed genes (DEGs) specific to R4VPL3-1 treatment. Of these, 191 genes were upregulated and 125 downregulated upon treatment with R4VPL3-1 (Supplementary Table S1). Pathway analysis using the Kyoto Encyclopedia of Genes and Genomes (KEGG) and Ingenuity Pathways Analysis (IPA) identified several upregulated pathways in response to R4VPL3-1 treatment. Among these pathways are the unfolded protein stress response, HIF-1α, and Estrogen signaling. Remarkably, among these upregulated pathways were also cell-death signatures of ferroptosis and necroptosis (Fig. 4C).

Ferroptosis is a form of regulated cell death characterized by iron-dependent lipid peroxidation and an increase in free cellular iron [43–47]. To determine whether R4VPL3-1 induces lipid peroxidation, a hallmark of ferroptosis, we treated A375R cells with 3 μM of R4VPL3-1, or 3 μM R4VPL3-1 along with the ferroptosis inhibitor 10 μM Ferrostatin-1 (Ferr-1; [44]). We used an established marker for lipid peroxidation, 4-Hydroxynonenal (4HNE), a major product of lipid peroxidation, and we used α-4HNE antibody to monitor its accumulation. We observed that R4VPL3-1 induced lipid peroxidation, as evident by positive 4HNE immuno-staining, which was inhibited by pre-treatment with 10 μM Ferr-1 (Fig. 4D; [44]). Moreover, we visualized live lipid peroxidation using the BODIPY-581/591-C11™ sensor. Upon lipid peroxidation, the C11-sensor shifts from a non-oxidized state (red) to an oxidized state (green). As shown in Fig. 4E, in control (DMSO) treated cells, the sensor is in its non-oxidized state, and the cells are mostly red, exhibiting only minimal oxidized signal (green). Upon exposure to 100 μM Cumene hydroperoxide, a known lipid-oxidation agent, the sensor molecules shift to green. Likewise, treatment with 3 μM R4VPL3-1 resulted in a similar and significant spectral shift of the sensor molecules that was not observed upon pre-treatment with 10 μM Ferr-1 (Fig. 4E and quantified in 4F). Collectively, these results suggest that R4VP3L-1 induces lipid peroxidation and ferroptosis.

To further establish that R4VPL3-1 induced ferroptotic cell death and test for potential involvement of additional cell death pathways that may mediate R4VPL3-1-induced cell death, we pre-treated A375R melanoma cells either with DMSO (control), or with Z-VAD-FMK (pan-caspase inhibitor), chloroquine (lysosomal inhibitor), or Necrostatin (a necroptosis inhibitor), or Ferr-1 (ferroptosis inhibitor), and subsequently treated the cells with 3μM R4VPL3-1. Indeed, control (DMSO), Necrostatin, Z-VAD, or chloroquine failed to inhibit R4VPL3-1-induced cell death. However, Ferr-1, or the iron chelator deferasirox (DFX), completely prevented R4VPL3-1-induced cell death (Fig. 4G–H, Supplementary Fig. S5). Along this line we observed that while exposure of the cells to 2 μM sterosproine (STS), induced the cleavage of PARP that is a hallmark of apoptosis, R4VPL3-1 did not cause cleavage of PARP (Fig. 4K, L). Note that the entire experiment was performed in A375R cells, which are resistant to PLX4032 treatment, and under 2 μM PLX4032 treatment in the medium. PLX4032 is an analogue of Vemurafenib, which is an established inhibitor of necroptosis [48]. We concluded that R4VPL3-1 induces ferroptotic cell death and likely no other cell death pathways.

**Fig. 5:**
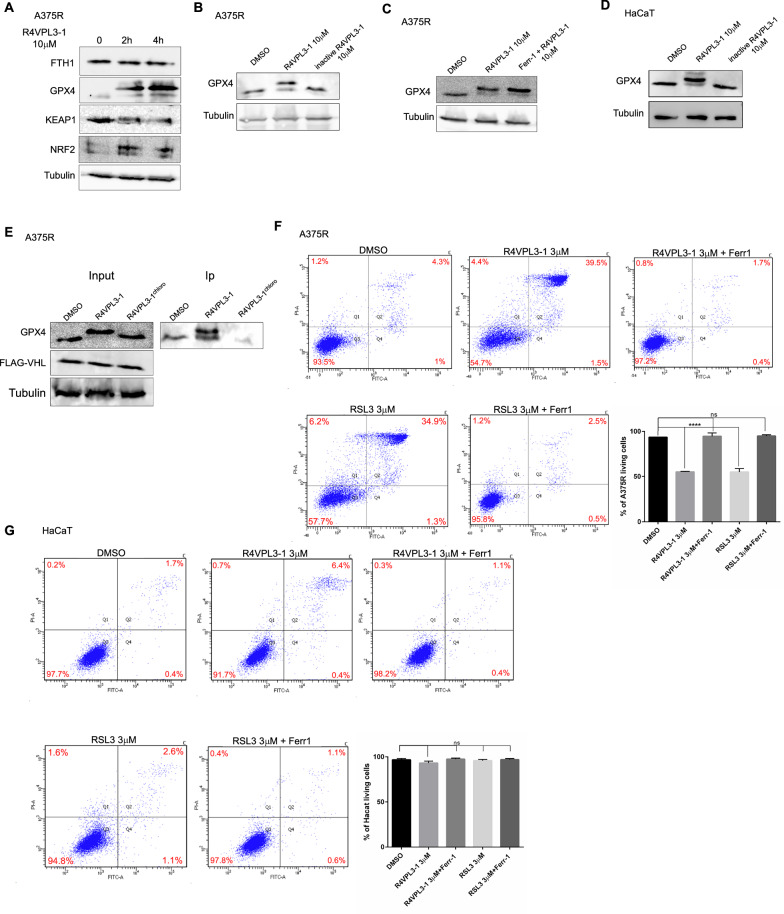
The anti-ferroptotic selanoprotein GPX4 is targeted by R4VP3L-1. **A** Western blot analysis of protein extracts derived from A374R cells treated with R4VPL3-1 for the indicated times and antibodies. Note the increased protein level and shift of GPX4 upon R4VPL3-1 treatment (see text for details) (**B–D**). Treatment of A375R cells with R4VP3-L1, but not inactive R4VP3-L1 results in a slower migrating form of GPX4 in A375R (**B**), and HaCat cells (**C**), and is not prevented by Ferr-1 (**D**). **E** VHL bind to endogenous GPX4 and to modified GPX4 via R4VPL3-1, does not bind GPX4 in the presence of R4VPL3-1^chloro^ inactive compound in an immunoprecipitation assay in A375R cells. **F**, **G** FACS analysis using PI and annexin 5, RSL-3, an inhibitor of GPX4 and selanoproteins, induces cell death of A375R that is inhibited by Ferr-1 (**F**), but does not induce cell death of HaCat cells (**G**). In both (**F**, **G**), representative experiments and quantification of three biological repeats are shown.

The significant ferroptotic stress imposed by R4VPL3-1 concomitantly resulted in a failed counter-activation of ferroptosis-inhibiting genes at transcriptional and protein levels. We observed an increased level of the nuclear factor erythroid-related factor 2 (NRF2/NFE2L2), a major anti-ferroptotic transcription factor [49]. This increase was observed along with a reduction in protein levels of the Kelch domain E3 ligase Keap1, which catalyzes the ubiquitin-dependent degradation of NRF2, which is also degraded by RNF4 (Fig. 5A; [50–52]). In addition, we observe an accumulation of selanoprotein enzymes that reduce oxidative stress and are NRF2 targets, genes, inhibit ferroptosis. Of specific interest is Glutathion Peroxidase 4 (GPX4), a major anti-ferroptotic selanoprotein protein (Fig. 5, Supplementary Table 1; [44, 53–56]). However, despite the increase in the anti-ferroptotic response, R4VPL3-1 induced rapid ferroptotic cell death. In this regard, we observed that the protein levels of GPX4 in cells treated with R4VPL3-1 increased and exhibited a slower migrating form in the SDS-PAGE. Treatment with inactive R4VPL3-1 lacking the Chloro-atom (R4VPL3-1^chloro^) that was biologically inactive and did not result in increased levels of GPX4, and neither in a molecular shift. Interestingly, this shift in molecular-wight was observed also in HaCat cells and was not prevented by Ferr-1 (Fig. 5B–D). Indeed, in a binding assay in cells R4VPL3-1, but not the inactive mutant R4VPL3-1^chloro^ bind to modified GPX4 (Fig. 5E, and see discussion). Moreover, the ferroptotic cell death induced by R4VPL3-1 was highly similar to the one induced by RSL3, a well-known ferroptosis inducer [57]; Both compounds induced ferroptotic cell death that is inhibited by Ferr-1, and both R4VPL3-1, and RSL3 did not induce cell death of HaCaT cells (Fig. 5F, G). Taken together, we speculate that the ferroptosis-inducing activity of R4VPs requires the combined action of R4VPL3-1 that co-inactivates anti-ferroptotic selanoproteins such as GPX4, along with the elimination of RNF4 and the anti-ferroptotic E3 ligase VHL.

### Molecular determinant for R4VPL3-1 selectivity

**Fig. 6:**
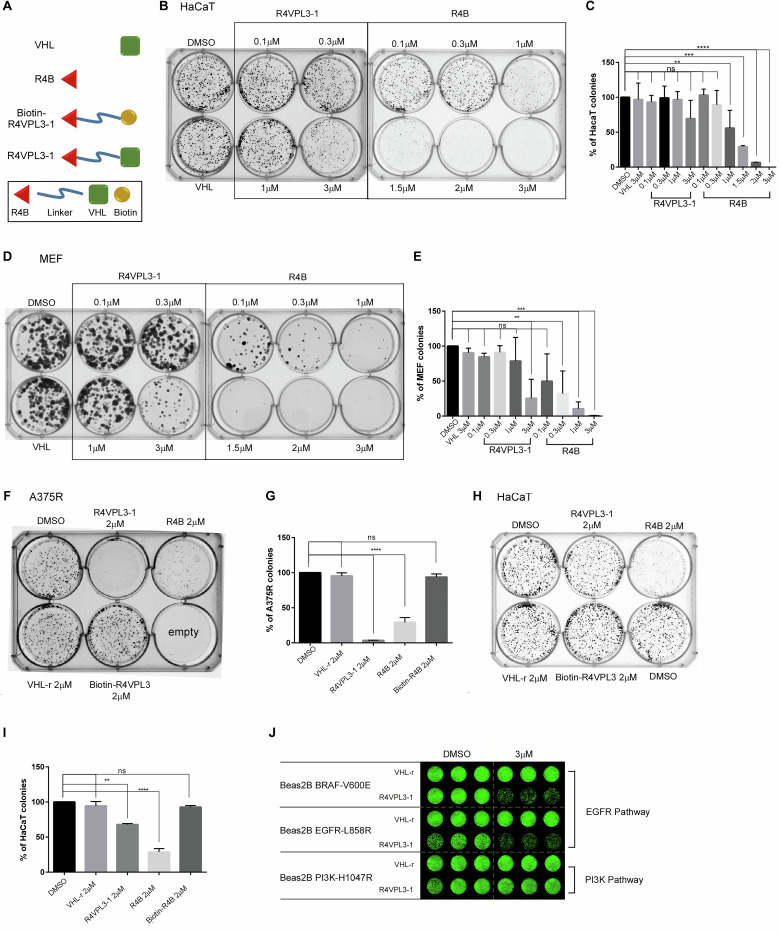
Structural determinants of R4VPL3-1 and selectivity involved in its differential anti-cancer activity. **A** Schematic diagram of R4VPL3-1-related compounds; R4B, RNF4 binding moiety. Testing the activity of R4VPL3-1-related compounds using SFA toward non-tumorigenic HaCaT cells (**B**, **C**) and MEFs (**D**, **E**). **B**, **D** are representative experiments. **F–I** SFA; Biotin-R4VPL3-1 (R4VPL3-1, where the VHL-r moiety was replaced with biotin), has no anti-cancer activity towards A375R (**F**, **G**) or HaCat (**H**, **I**). **J** Live Cell proliferation assays using a-LamA/C of the indicated transformed epithelial lung cells (see methods). R4VPL3-1 has selective anti-proliferative activity against Beas2B lung cells transformed with either BRAF^V600C^ or EGFR^L858R^ activating mutations, but not PI3K^H1047R^ mutation. Statistical analysis: 1way Anova Dunnett’s multiple comparisons test (**C**, *n* = 3; **E**, *n* = 3; **G**, *n* = 2; **I**, *n* = 2); Significance: ****p < 0.0001, ***p < 0.001, **p < 0.0, ns no-significance.

We were intrigued by the differential effects of R4VPs that induced cell death of cancer cells but did not affect non-tumorigenic skin keratinocyte HaCat cells or primary MEFs. Therefore, we tested for inhibition of SFA upon treatment with the different R4VPL3-1 components, as well as R4VPL3-1, where we replaced the VHL-r moiety with Biotin (Biotin-R4VPL3-1, Fig. 6A). We observed that R4B, the RNF4 binding moiety, had no selectivity and inhibited SFA of both non-tumorigenic HaCat cells and primary MEFs as well as cancer cells. In contrast, R4VPL3-1 had selective and potent activity toward cancer cells but had no activity against non-tumorigenic cells at concentrations ranging from 0.1 μM to 2 μM (Fig. 6B–E). Moreover, Biotin-R4VPL3-1 was an inactive compound with no activity against both cancer cells and non-tumorigenic cells (Fig. 6F–I). Likewise, replacing the VHL-binding moiety with Cerbelon-recruiter in the original R4VP compound had no anti-cancer activity (not shown), suggesting that the VHL moiety is critical for R4VPs’ anti-cancer activity.

Remarkably, we discovered that R4VPL3-1 exhibits differential activity against cancer cells depending on the oncogenic driver mutation used to induce cell transformation. In this experiment, we used a human tracheal epithelial cell line, BEAS-2B, that was retrovirally transformed with activated oncogenes: BRAF^V600A^, EGFR^L858R^, or PI3K^H1017R^ [41]. R4VPL3-1 inhibited the proliferation of BRAF^V600A^, EGFR^L858R^, transformed BEAS-2B cells but did not inhibit the proliferation of PI3K^H1017R^ transformed cells (Fig. 6J). This is likely due to intrinsic resistance to ferroptosis that is induced by PI3K~mTor signaling ([58]; see discussion). Collectively, these results suggest that all of the R4VP3L-1 modules are necessary to form a selective anti-cancer compound that is highly active against ferroptosis-sensitive cancer cells. Interestingly, we observed that both EGFR pathway- and PI3K-transformed cells, are “addicted” to RNF4 and VHL; We failed to genetically eliminate either RNF4, or VHL, or both using CRISPER/CAS9 gene editing, and all the surviving cells were “escapers” expressing both RNF4 and VHL (Supplementary Fig. S3).

### Testing of R4VPL3-1 anti-cancer activity in patient-derived primary sarcoma cells

**Fig. 7:**
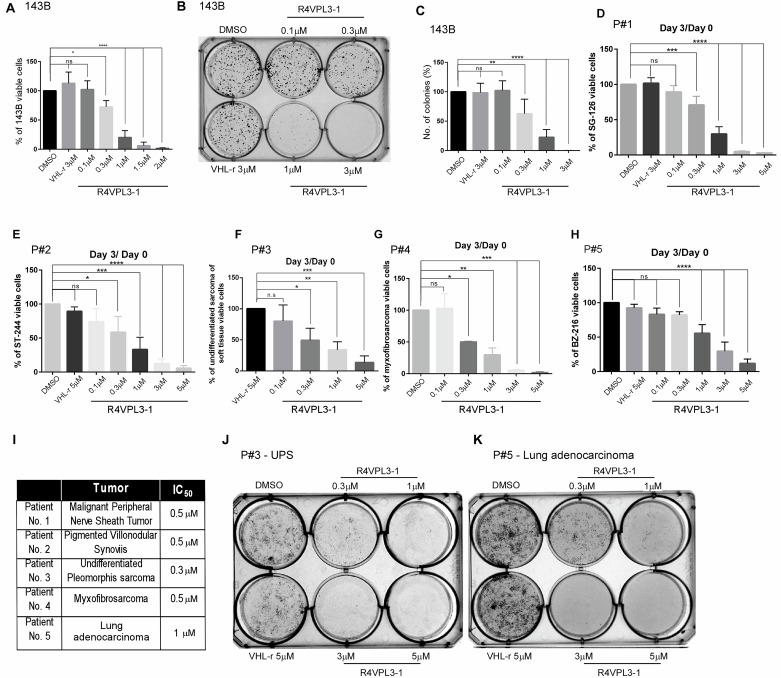
R4VPL3-1 is active towards sarcoma cells and patients-derived primary tumor cells. R4VPL3-1 inhibits cell proliferation (**A**) and SFA (**B**, **C**) of 143B cells, a highly metastatic human sarcoma cell line. **D**–**H** R4VPL3-1, but not DMSO, or the VHL-r, inhibits cell proliferation of patient-derived primary tumor cells in a dose-dependent manner. **I** Table summarizing IC_50_ of R4VPL3-1 in different patient-derived cells. Patients #1–4 are sarcoma tumor cells and patient #5 is a lung adenocarcinoma tumor. **J**, **K** R4VPL3-1 inhibits SFA of undifferentiated polymorphic sarcoma (UPS) (**J**), and lung adenocarcinoma tumor cells (**K**). Statistical analysis: One-way Anova Dunnett’s multiple comparisons test (**A**, *n* = 3; **C**, *n* = 4; **D**, *n* = 4; **E**, *n* = 3; **F**, *n* = 3, **G**, *n* = 2; **H**, *n* = 3); Significance. ****p < 0.0001, ***p < 0.001, **p < 0.01. ns non-significant.

Sarcomas of multiple types present a clinical challenge [59–61]. We previously observed that RNF4 is essential for sarcoma tumorigenesis, and that high levels of RNF4 are observed across multiple sarcoma entities and are associated with poor sarcoma patient survival [11]. Moreover, induction of ferroptosis emerges as a potential therapeutic venue in sarcomas [62–64]. Therefore, we decided to test the impact of R4VPs on the metastatic human sarcoma cell line 143B. R4VPL3-1 treatment of these cells resulted in the inhibition of proliferation and SFA (Fig. 7A–C). Remarkably, R4VPL3-1 was potent towards primary untreated patient-derived sarcoma and lung tumor cells isolated during onco-surgical resections, inhibiting proliferation and sphere formation in culture with IC_50_ ~ 0.5μM of multiple sarcoma entities (Fig. 7D–K). Thus, R4VPL3-1 is highly active against sarcoma cells, including diverse primary patients-derived sarcoma tumor cells.

## Discussion

We developed ferroptosis-inducing molecules termed R4VPs that eliminated multiple cancer cell types, including RTKi-resistant human melanoma, primary patient-derived sarcoma, and metastatic lung cancer cells, depending on the tumor-driving mutation. In contrast, non-tumorigenic cell lines and primary MEFs are not affected by R4VPs. R4VPs induce the elimination of RNF4 and its stabilized, phosphorylated-oncoproteins as well as the E3 ligase VHL and modified the anti-ferroptotic selanoprotein GPX4. R4VPL3-1 induced-ferroptosis is fully inhibited by the ferroptosis inhibitor Ferr-1 and the iron chelator DFX, but not inhibitors to other cell death pathways. Thus, confirming the molecular mechanism by which R4VPs eliminate aggressive tumor cells.

We used CCW16, an RNF4-binding moiety [39], as a starting point for a focused small-molecule screen and identified an RNF4 binder with higher affinity (R4B), which served as a building block for developing more potent R4VPs. Previous mass spectrometry analysis suggested that TRH1-23, an early version of CCW16, reacts with Cys132 and Cys135 residues within the RING domain of RNF4 [39]. However, the binding of R4VPL3-1 to RNF4 is different, as it reacts with Cys 51 and Cys 91 outside RNF4’s RING domain. This was further confirmed by mutating Cys 132 and 135, as these had only minimal impact on R4VPL3-1 binding to RNF4 (Müller~Orian personal communication).

R4VPs exhibit selective activity, eliminating cancer cells but not non-tumorigenic cells. We observed that R4VPs selectively induce the degradation of VHL, RNF4 and its stabilized oncoproteins in cancer cells, but not in non-tumorigenic HaCaT cells. The selectivity may stem from multiple reasons; The ability of a POI to effectively undergo ubiquitination is dependent on the substrate’s availability and the activity of E3/E2 proteins. It is possible that the low levels of RNF4 protein present in HaCaT cells may be less available, as it may be largely associated with nuclear PML bodies or bound to SUMOylated proteins. While VHL protein levels are high in HaCaT cells, other molecules may be required for its ubiquitination activity towards the specific POI that are absent in these cells. Interestingly, E3 ligases display heterogeneous, tissue- and cell-type-specific expression patterns and activity that may determine PROTAC efficiency [65].

The ability of a given PROTAC to force the degradation of a POI is also greatly dependent on the function of 26S proteasome. It has been shown that the proteasome is significantly more active in tumor cells than in normal or immortalized epithelia [66, 67]. Elevated overall proteasome activity and specific expression of proteasome subunits (PSMB3, PSMA4, PSMD4) were observed across multiple carcinomas relative to normal tissues [66]. Likewise, TCGA analyses showed that proteasome catalytic subunits are overexpressed in 8/13 tumor types compared to matched non-transformed tissue [68]. Thus, it is possible that SCC1 or A375R skin cancer and melanoma cells clear ubiquitinated proteins more rapidly than non-tumorigenic HaCaT keratinocytes.

We observed that the VHL-r moiety was essential for R4VPL3-1 anti-cancer activity, as its replacement with biotin, or with the well-established recruiter moiety of the E3 Cerbelon [30], resulted in inactive compounds. The VHL moiety may be required for the elimination of VHL, which itself is a strong anti-ferroptotic protein, or for the ability of R4VPs to bind, modify or eliminate other POIs that are suppressors of ferroptotic cell death. One such protein is GPX4 that is a potent anti-ferroptotic selanoproteins that is covalently bound and modified by R4VPL3-1. Interestingly, GPX4 was modified, but not degraded, by R4VP4 in both A375R and HaCaT cells. However, HaCaT cells are not sensitive to R4VPs, and R4VPL3-1 does not induce the degradation of either RNF4 or VHL in these cells. Taken together, the selective lethality observed in multiple cancer cells may require the co-elimination of RNF4, VHL, along with the inactivation of GPX4 or similar selanoproteins. A proteomic approach to identify such additional R4VPs targets is ongoing and is outside the scope of this manuscript.

While R4VPs induced the degradation of RNF4, it also leads to the elimination of VHL in cancer cells. This is different from the case of a VHL-based PROTAC targeting the oncogenic E3 ubiquitin ligase MDM2 in triple-negative breast cancer [69]. In this regard, it was also shown that VHL expression in RCC cells prevented ferroptosis induction [70]. However, our attempt to inhibit the anti-cancer activities of R4VPs by inhibiting the ubiquitin pathway using the E1 inhibitor TAK-243 was not successful due to significant and rapid cell toxicity upon co-treatment. Similarly, we observed that inhibition of RNF4 or VHL elimination via R4VP3L-1 upon proteasome inhibition was challenging, albeit the observation that both RNF4 and VHL accumulate in cells treated with the proteasome inhibitor MG132. Considering these limitations, R4VPs cannot be regarded as a classical PRTOACs. However, R4VPs are potent ferroptosis inducers (FINs) with selective anti-cancer activity.

Defining target specificity and selectivity is a central question in the development of small-molecule inhibitors and protein degraders [71]. We have shown that the expression of RNF4^DM^, which does not bind to R4VPL3-1; did not prevent R4VPL3-1-induced ferroptosis. R4VPs sensitive cells are addicted to RNF4 and VHL (this work [10, 11]). Yet, due to technical difficulties we were not able to perform the optimal experiment, in which the endogenous RNF4 is eliminated either by CRISPR/CAS9 gene editing or by 3’ UTR-directed shRNA and replaced by the co-expression of RNF4^DM^ in A375R. As RNF4 functions as a dimer [72], we therefore cannot formally rule out the possibility that the RNF4^DM^ expressed in these cells dimerizes with wild-type RNF4 that binds to R4VPL3-1, leading to the degradation of the RNF4/RNF4^DM^ heterodimer and VHL.

Ferroptosis is a cell death pathway with distinct molecular and histopathological features [43]. It is intrinsically inhibited by NRF2, the key antioxidant transcription factor that is targeted for degradation by the ubiquitin ligases Keap1 and RNF4 [50–52]. It is also inhibited by multiple enzymes and metabolic pathways, such as Cys and Cys/Glu transporters, which increase the activity of antioxidant enzymes and reduce free iron, as well as enzymes that minimize lipid peroxidation, such as GPX4 [43, 53–56]. Indeed, the strong ferroptotic induction by R4VPL3-1 induced a transcriptional response and upregulation of anti-ferroptotic proteins similar to those reported in other studies [73]. Yet these were not sufficient to stall ferroptosis likely because of R4VPL3-1 inactivation of these proteins such as in the case of GPX4. Interestingly, we observed that VHL itself may bind to GPX4, and via R4VP3L-1 interacts with the modified form of GPX4. Both interactions are not observed in the R4VP3L-1^chloro^ inactive mutant. Yet, additional verification using purified R4VPs and GPX4 will further corroborate the direct nature of this interaction. Moreover, the biological importance of VHL ~ GPX4 interaction is still not clear along with a report for indirect connection between the two proteins in renal cell carcinomas [70]. We also tested the possibility that cell death results from compensatory hyper-activation and reductive stress response to oxidative stress, which may lead to cell death [74]. Yet, inhibition of a key enzyme in the antioxidant response such as aldehyde dehydrogenase ALDH3A1, using its inhibitor ALDH3A1-IN-3 did not inhibit R4VPL3-1 induced ferroptosis (Supplementary Fig. S5).

One hallmark of cancer cells is their ability to evade cell death by multiple mechanisms [1, 75]. The sensitivity to ferroptosis varies among cancer cells and is context-dependent [43]. In this regard, even within the same cell of origin, sensitivity to R4VPL3-1 depended strongly on the identity of the cancer-driving mutation used for cell transformation. Epithelial cells transformed with activated oncogenes within the EGFR pathway were susceptible to R4VPL3-1. However, a mutation that activated the oncogenic PI3K was resistant to R4VPL3-1. PI3K signaling via the mechanistic target of rapamycin (mTOR) pathway is well known to suppress ferroptosis by increasing the processing and activity of the ER-anchored transcription factor sterol regulatory element-binding protein (SREBP). In turn, SREBP induces the expression of stearoyl-CoA desaturase (SCD1), which enhances the production of ferroptosis-suppressing monounsaturated fatty acid (MUFA; [76, 77]). However, our attempt to sensitize BEAS-2B transformed cells to R4VPL3-1 by inhibiting SCD1 was not successful.

Thus, the differential activity of R4VPs may depend on the presence of R4VPs substrate(s) that inhibit ferroptosis, which these cancer cells are addicted to. Treatment with R4VPs likely results in their degradation and a shift in the balance between anti- and pro-ferroptotic cell states. Thus, as R4VPs anti-cancer activity stems from inducing ferroptosis, we predict that ferroptosis-resistant cells will resist R4VPs.

Mounting evidence suggests that inducing ferroptosis may be a potent strategy to initiate cancer cell death and specifically in sarcomas [47, 78, 79]. Ferroptosis can be induced genetically by the expression of ferroptotic inducer genes such as NCOA4, miR-129p, miR-672-3p, or by small molecules collectively termed ferroptosis inducers, FINs [for a comprehensive review regarding FINs, see ref. [79]]. Moreover, ferroptosis can be induced by a PROTAC, which induces the degradation of GPX4 [80]. However, the case of R4VPs-induced ferroptosis differs and likely involving inactivation of GPX4 via direct binding rather than its degradation. Moreover, while our study was under revision Gotthardt et al. described the development of EN219 a ferroptosis-inducing compound that share some structural similarities to R4VPs [81]. Importantly, R4VPs are biologically highly different from EN219; R4VPs harbor intrinsic cancer-selective activity, lead to the co-elimination of both RNF4 and VHL and modify GPX4, that all are features that are unique to R4VPs and are not observed in EN219.

Our study suggests that R4VPs are bona fide FINs that induce ferroptosis but not necroptosis, autophagy, or apoptosis. While R4VPs are potent against RTKi-resistant melanoma and multiple sarcoma entities, the full spectrum of R4VPL3-1 sensitive and resistant cancer entities awaits future studies. In this regard, sarcomas of different types, including bone and soft-tissue sarcomas, present a clinical challenge. Albeit extensive efforts, to date no significant improvement in patients’ survival, specifically in advanced metastatic disease, has been made [60, 61, 82]. Thus, our observation that R4VPL3-1 is effective in eliminating multiple patient-derived sarcoma entities is encouraging. Finally, our observation that R4VPs are potent ferroptotic inducers suggests that they have the potential to be effective for eliminating various aggressive cancer entities.

## Supplementary information

Supporting chemical SI

Supporting materials -materials and methods and figures

R4VPl3-1 RNA-seq gene list

## Data Availability

All data are available in the main text or the supplementary materials. The RNA-seq-data is available at: GSE284185.
