# Supplementary material for: Discovery of ferroptosis-inducing R4VP compounds for targeting aggressive cancers
Source: Oncogene. 2026 Jun 5;45(27):2727–42. doi: 10.1038/s41388-026-03829-2 (PMC13314539; doi:10.1038/s41388-026-03829-2)
Supplement: Supplementary file 2 — Supporting materials -materials and methods and figures [file 41388_2026_3829_MOESM2_ESM.pdf]

This file includes:

1. Figs. S1A, S1B, S2, S3, S4, S5, S6

Other Supplementary Materials for this manuscript include the following:

Data S1 Excel file: R4VPL3-1 regulated genes
Chemical SI

AVO et al. Figure S1A

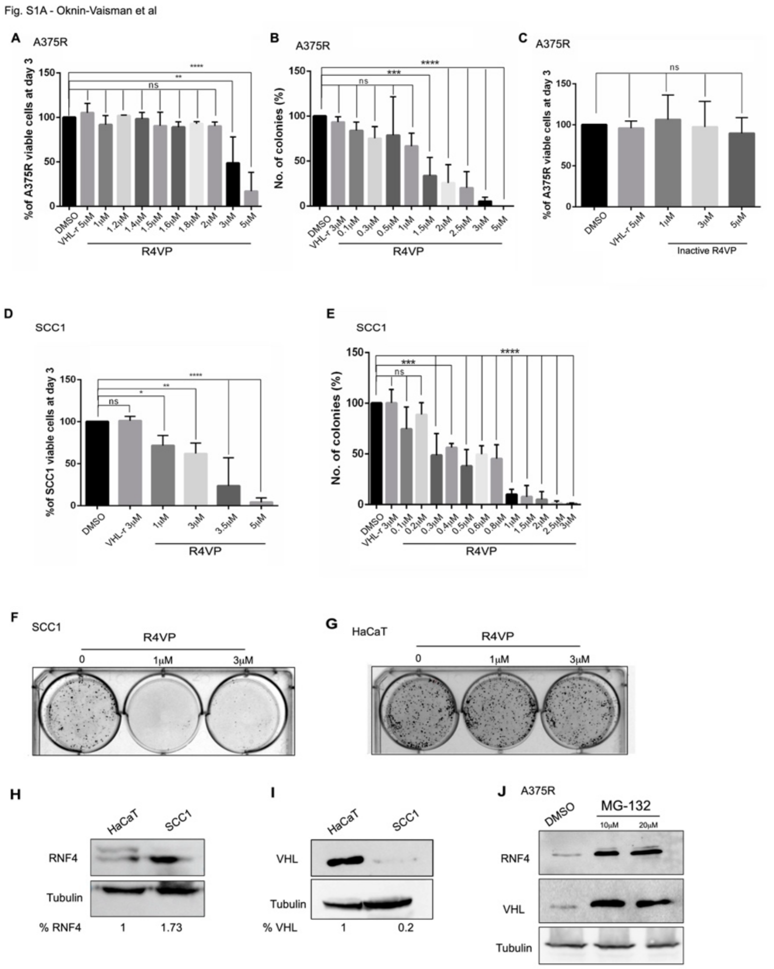

Figure S1A: R4VP force the degradation of RNF4 and reduces survival of cancer cells. (A,B) R4VP but not DMSO, or VHL-r, inhibits the proliferation (A) and SFA (B) of A375R melanoma cells. **(C)** Inactive R4VP lacking a critical Chloro atom has no impact on A375R cells proliferation . **(D)** R4VP treatment, but not DMSO or ,VHL-r, inhibits proliferation of human skin cancer cells SSC1. **(E-G)** R4VP inhibits SFA of SSC1 cells (E, F) but had no impact on non-tumorigenic human HaCat cell line (G). Cell proliferation was measured indirectly by ATP-Lite assay. In all experiments n=3 and ***= $p<0.001$ **=$p<0.01$,*=$p<0.1$, statistical analysis was performed using 1way Anova Dunnett's multiple comparisons test. **(H, I)** RNF4 (H) and VHL (I) protein levels in HaCat and SCC1 cells **(J)** RNF4 and VHL protein levels in A375R cells upon treatment with proteasome inhibitor MG132.

AOV et al. Figure S1B

Fig. S1B - Oknin-Vaisman et al

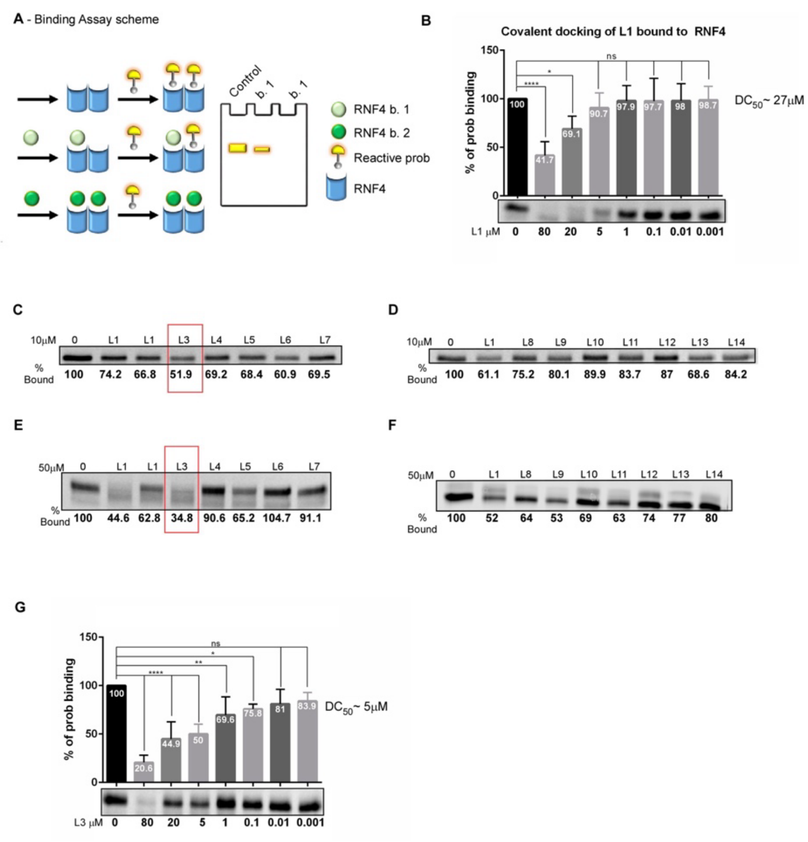

Figure S1B: Identification of an enhanced RNF4 binding moiety. We generated CCW16 - related compounds (L2-L14) and measured their ability to directly bind RNF4 purified from bacterially *in vitro* using a 5-TAMRIA-Iodoacetamide (T-I) labeling and displacement assay enabling quantitative visualization of RNF4 similar to (39). Using this assay, we determined that the binding of the original CCW16 molecule (termed L1) to RNF4 resulted in reduced signal in a dose-dependent manner, with a 50% binding inhibition concentration of ~27μM. We compared the binding of L1 to newly developed L1-related molecules L2-L14. Of the compound tested we identify L3 as an RNF4 binder with improved binding affinity with 50% T-I inhibition at ~5μM. **(A)** Schematic diagram of a direct 5-TAMRA-Iodacetmide dye displacement assay by the RNF4 binding moiety. **(B)** Upper panel: concentration dependent dye displacement by the L1, n=3, ***= p<0.001. Lower panel: Representative experiment. **(C-F)** Screening of TI-displacement of potential RNF4 binders at 10μM (C, D) and 50μM (E, F). **(G)** Upper panel: concentration dependent dye displacement by L3, an improved RNF4 binding moiety n=3, ****= p<0.0001. Lower panel is a representative experiment.

AOV et al. Figure S2

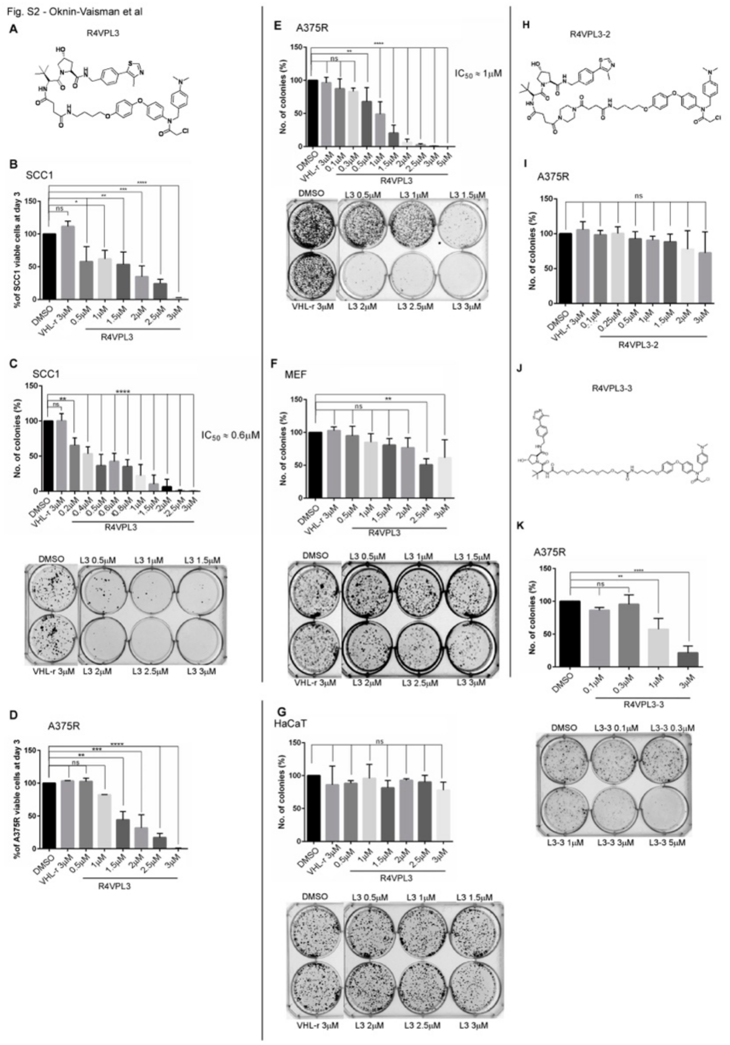

Figure S2: Anti-cancer activities of R4VPL3, R4VPL3-2 and R4VPL3-3. Based on L3, we synthesized R4VPL3, a using L3 as the RNF4 binder that was more potent than L1 and retained its selective activity against cancer cells but had no impact on proliferation or SFA of MEFs. **(A)** Structure of R4VPL3 **(B-F)** Dose-dependent effect of R4VPL3 on viability and SFA of SCC1 cells (B, C) and A375R melanoma (D, E) cells. Lower panels in C and E are representative experiments. Cell proliferation was measured indirectly by MTT). **(F, G)** R4VPL3 had minimal or no impact on SFA of non-tumorigenic cells; Mouse embryonic fibroblasts (MEFs; G)) and HaCaT (H). **(H)** Structure of R4VPL3-2 **(I)** R4VPL3-2 had no impact on SFA of human melanoma A375R cells. **(J)** Structure of R4VPL3-3. **(K)** R4VPL3-3 inhibited SFA of A375R cells and lower panels is a representative experiment. In all experiments n=3 and ***=p<0.001, **=p<0.01. Statistical analysis was performed using One-way Anova Dunnett's multiple comparisons test.

AOV et al. Figure S3

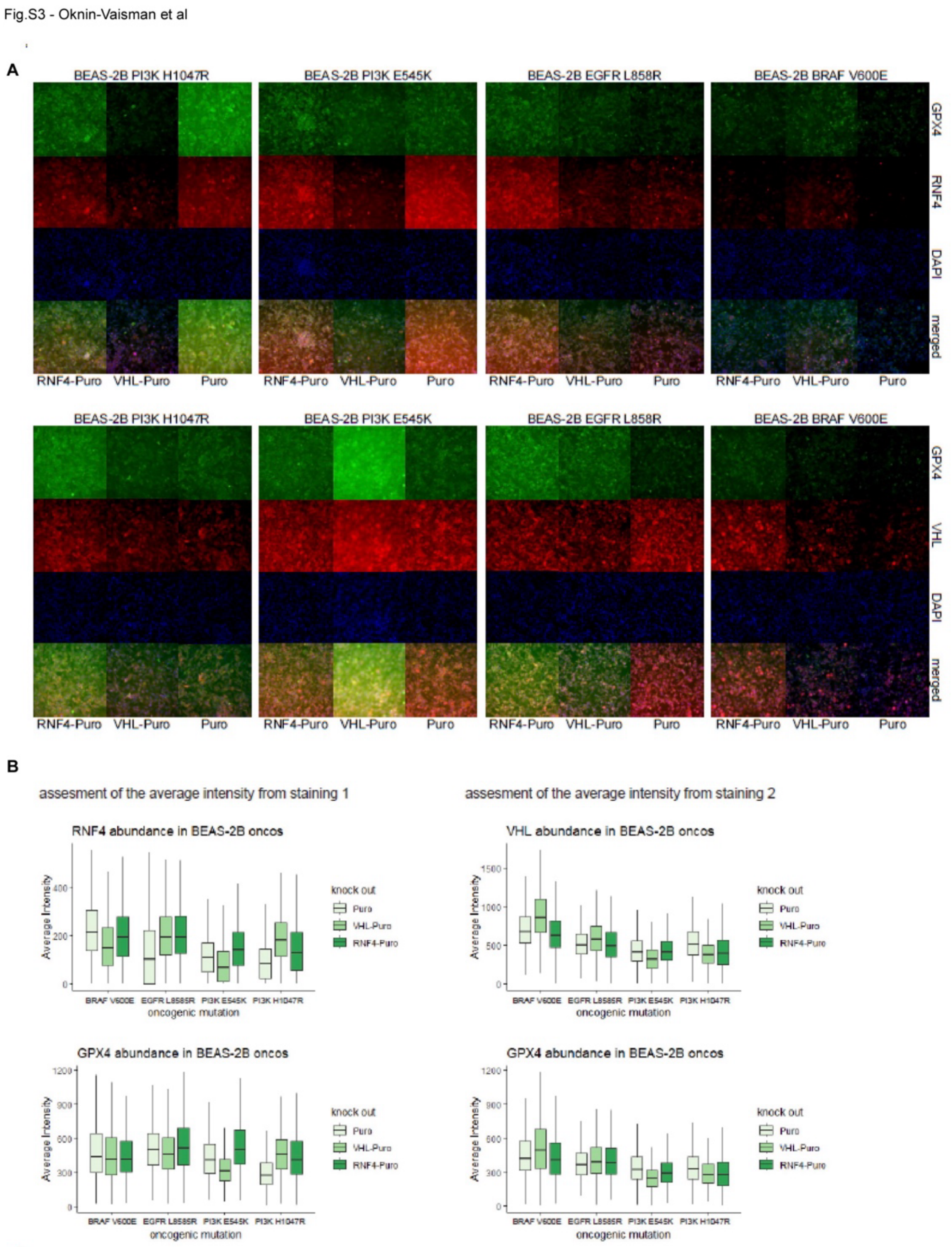

Figure S3: VHL and RNF4 are essential for BEAS-2B transformed cancer cell survival. Immunofluorescence microscopy analysis of RNF4, VHL and GPX4 abundance in BEAS-2B onco (PIK^{H1047R}, PIK^{E545K}, EGFR^{L858R} and BRAF^{V600E}, respectively) after targeting of either RNF4 or VHL by CRISPR, followed by puromycin selection. Puro indicates control sgRNA transfected BEAS treansformed cancer cells. Nuclear DNA is visualized with DAPI.

(A) Representative immunofluorescence microscopy images are depicted. **(B)** Analysis of the average immunofluorescence intensity visualised by RStudio.

AOV et al. Figure S4

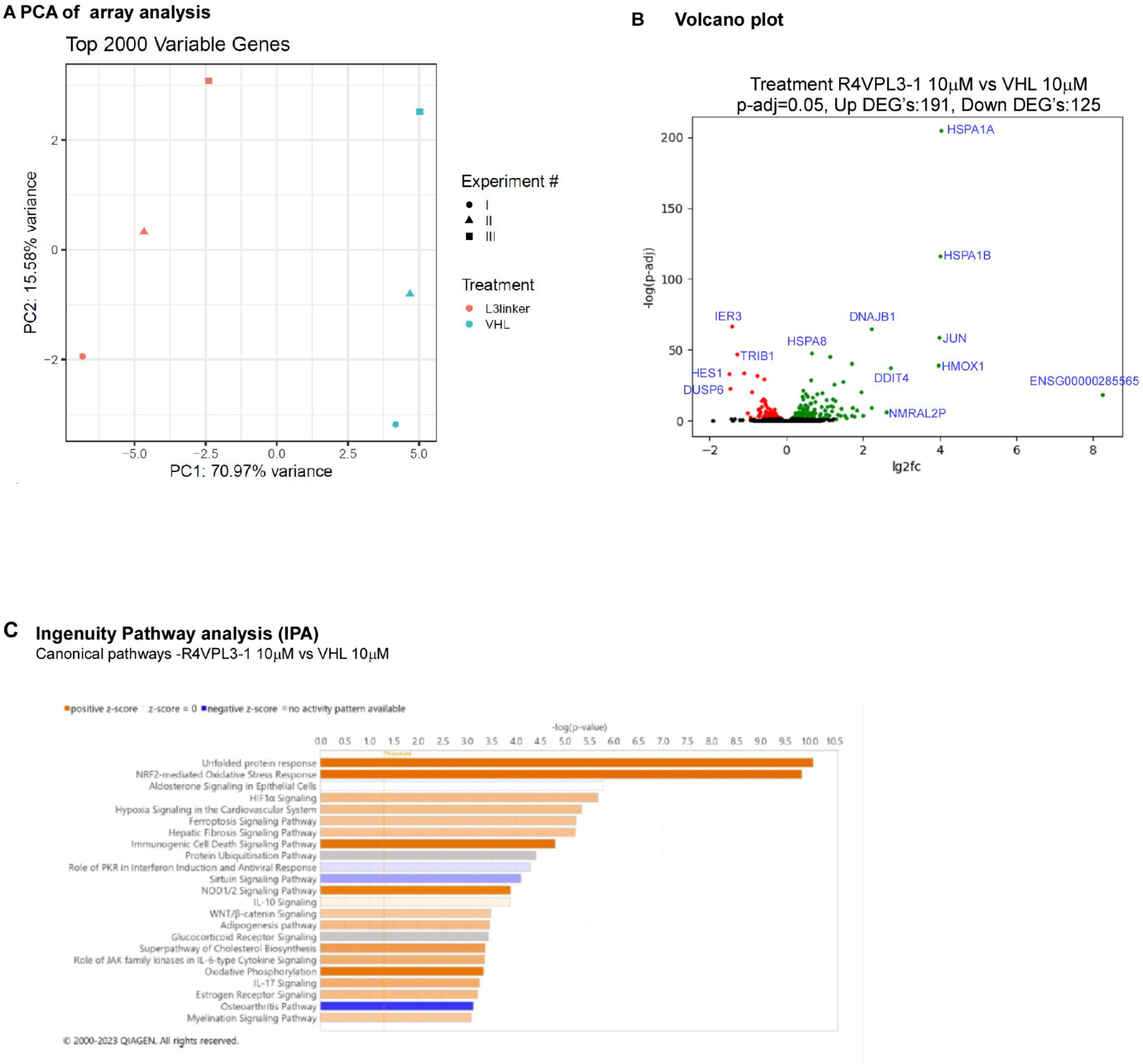

Figure S4: Impact of R4VPL3-1 on gene expression. RNA-Seq results comparing changes in gene expression signature between human melanoma A375R cells treated with 10μM of VHL-r or R4VPL3-1. **(A)** Three principal components (PCA) analysis of three experimental biological repeats. **(B)** Volcano plots of changes in gene expression here red circles indicates down regulated gene and green circles indicates upregulated genes, see methods for experimental and analysis details. **(C)** Top pathway identified using IPA analysis. Orange colored are upregulated pathways and blue are downregulated.

AOV et al. Figure S5

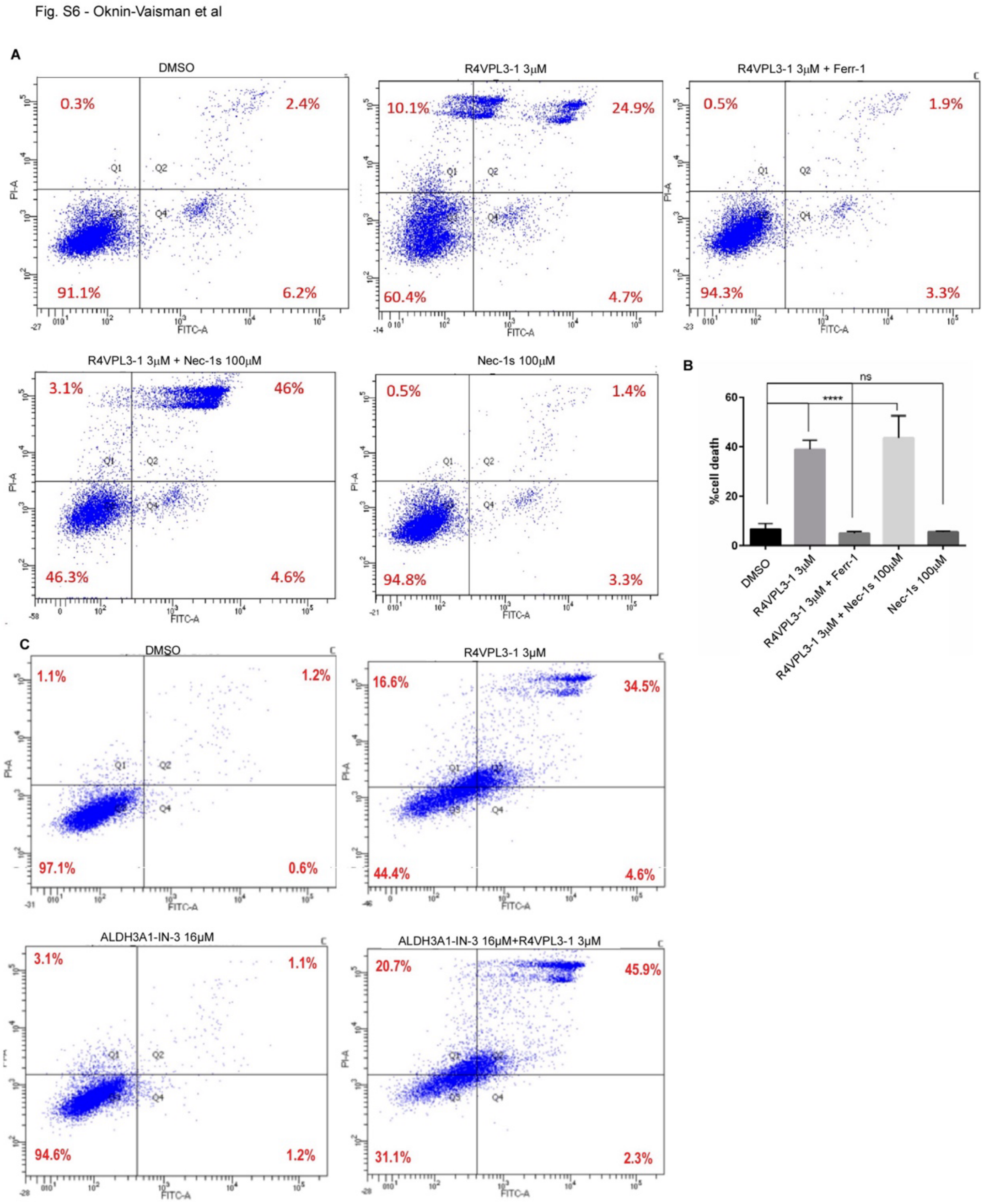

Figure S5 : R4VPL3-1 induced cell death is inhibited by Ferr-1 but not the necroptosis Nec 1 or LDH3A1 inhibitors. **(A)** Representative FACS images analysis of cell death using propidium iodide and Annexin V-FITC. Cells were treated with DMSO, R4VPL3-1, R4VPL3-1 and the indicated compounds or the indicated compounds alone (that were added 1h prior to R4VPL3-1 treatment. Unlike Ferr-1, the Necroptosis inhibitor, Nec-1 or the ALDH1 inhibitor did not prevent R4VPL3-1 induced cell death. **(B)** Quantification of 3 independent biological repeats. ****= P<0.0001.

S6.Sequence of sgRNA guide VECTOR:

The target vector pAAV sgRNAXhoI-EFS-SpCas9-P2A-Puro (sequence available in this paper) was generated using a gBlock (IDT) containing the EFS short promoter, a P2A cleavage site and the Puromycin resistance gene.
Vector sequence:

5′-TCACCGAAACGCGCGAGACGAAAGGGCCTCGTGATACGCCTATTTTTATAGGTTAATGTCATGATAATAATGGTTTCTTA
GACGTCAGGTGGCACTTTTCGGGGAAATGTGCGCGGAACCCCTATTTGTTTATTTTTCTAAATACATTCAAATATGTATC
CGCTCATGAGACAATAACCCTGATAAATGCTTCAATAATATTGAAAAAGGAAGAGTATGAGTATTCAACATTTCCGTGTC
GCCCTTATTCCCTTTTTTGCGGCATTTTGCCTTCCTGTTTTTGCTCACCCAGAAACGCTGGTGAAAGTAAAAGATGCTGA
AGATCAGTTGGGTGCACGAGTGGGTTACATCGAACTGGATCTCAACAGCGGTAAGATCCTTGAGAGTTTTCGCCCCGAAG
AACGTTTTCCAATGATGAGCACTTTTAAAGTTCTGCTATGTGGCGCGGTATTATCCCGTATTGACGCCGGGCAAGAGCAA
CTCGGTCGCCGCATACACTATTCTCAGAATGACTTGGTTGAGTACTCACCAGTCACAGAAAAGCATCTTACGGATGGCAT
GACAGTAAGAGAATTATGCAGTGCTGCCATAACCATGAGTGATAACACTGCGGCCAACTTACTTCTGACAACGATCGGAG
GACCGAAGGAGCTAACCGCTTTTTTGCACAACATGGGGGATCATGTAACTCGCCTTGATCGTTGGGAACCGGAGCTGAAT
GAAGCCATACCAAACGACGAGCGTGACACCACGATGCCTGTAGCAATGGCAACAACGTTGCGCAAACTATTAACTGGCGA
ACTACTTACTCTAGCTTCCCGGCAACAATTAATAGACTGGATGGAGGCGGATAAAGTTGCAGGACCACTTCTGCGCTCGG
CCCTTCCGGCTGGCTGGTTTATTGCTGATAAATCTGGAGCCGGTGAGCGTGGGTCTCGCGGTATCATTGCAGCACTGGGG
CCAGATGGTAAGCCCTCCCGTATCGTAGTTATCTACACGACGGGGAGTCAGGCAACTATGGATGAACGAAATAGACAGAT
CGCTGAGATAGGTGCCTCACTGATTAAGCATTGGTAACTGTCAGACCAAGTTTACTCATATATACTTTAGATTGATTTAA
AACTTCATTTTTAATTTAAAAGGATCTAGGTGAAGATCCTTTTTGATAATCTCATGACCAAAATCCCTTAACGTGAGTTT
TTGTTCCACTGAGCGTCAGACCCCGTAGAAAAGATCAAAGGATCTTCTTGAGATCCTTTTTTTCTGCGCGTAATCTGCTG
CTTGCAAACAAAAAAACCACCGCTACCAGCGGTGGTTTGTTTGCCGGATCAAGAGCTACCAACTCTTTTTCCGAAGGTAA
CTGGCTTCAGCAGAGCGCAGATACCAAATACTGTTCTTCTAGTGTAGCCGTAGTTAGGCCACCACTTCAAGAACTCTGTA
GCACCGCCTACATACCTCGCTCTGCTAATCCTGTTACCAGTGGCTGCTGCCAGTGGCGATAAGTCGTGTCTTACCGGGTT
GGACTCAAGACGATAGTTACCGGATAAGGCGCAGCGGTCGGGCTGAACGGGGGGTTCGTGCACACAGCCCAGCTTGGAGC
GAACGACCTACACCGAACTGAGATACCTACAGCGTGAGCTATGAGAAAGCGCCACGCTTCCCGAAGGGAGAAAGGCGGAC
AGGTATCCGGTAAGCGGCAGGGTCGGAACAGGAGAGCGCACGAGGGAGCTTCCAGGGGGAAACGCCTGGTATCTTTATAG
TCCTGTCGGGTTTCGCCACCTCTGACTTGAGCGTCGATTTTTGTGATGCTCGTCAGGGGGGCGGAGCCTATGGAAAAACG
CCAGCAACGCGGCCTTTTTACGGTTCCTGGCCTTTTGCTGGCCTTTTGCTCACATGTCCTGCAGGCAGCTGCGCGCTCGC
TCGCTCACTGAGGCCGCCCGGGCGTCGGGCGACCTTTGGTCGCCCGGCCTCAGTGAGCGAGCGAGCGCGCAGAGAGGGAG
TGGCCAACTCCATCACTAGGGGTTCCTGCGGCCGCACGCGTGATATCGGATCCGTTAACTCTAGATTTAAAGGTACCGAG
GGCCTATTTCCCATGATTCCTTCATATTTGCATATACGATACAAGGCTGTTAGAGAGATAATTAGAATTAATTTGACTGT
AAACACAAAGATATTAGTACAAAATACGTGACGTAGAAAGTAATAATTTCTTGGGTAGTTTGCAGTTTTAAAATTATGTT
TTAAAATGGACTATCATATGCTTACCGTAACTTGAAAGTATTTCGATTTCTTGGCTTTATATATCTTGTGGAAAGGACGA
AACACCTCGAGTTTTAGAGCTAGAAATAGCAAGTTAAAATAAGGCTAGTCCGTTATCAACTTGAAAAAGTGGCACCGAGT
CGGTGCTTTTTTAGGCCTGGTCTTGAAAGGAGTGGGAATTGGCTCCGGTGCCCGTCAGTGGGCAGAGCGCACATCGCCCA
CAGTCCCCGAGAAGTTGGGGGGAGGGGTCGGCAATTGAACCGGTGCCTAGAGAAGGTGGCGCGGGGTAAACTGGGAAAGTG
ATGTCGTGTACTGGCTCCGCCTTTTTCCCGAGGGTGGGGGAGAACCGTATATAAGTGCAGTAGTCGCCGTGAACGTTCTT
TTTCGCAACGGGTTTGCCGCCAGAACACAGGCGTACGGCCACCATGGACTATAAGGACCACGACGGAGACTACAAGGATC
ATGATATTGATTACAAAGACGATGACGATAAGATGGCCCCAAAGAAGAAGCGGAAGGTCGGTATCCACGGAGTCCCAGCA
GCCGACAAGAAGTACAGCATCGGCCTGGACATCGGCACCAACTCTGTGGGCTGGGCCGTGATCACCGACGAGTACAAGGT
GCCCAGCAAGAAATTCAAGGTGCTGGGCAACACCGACCGGCACAGCATCAAGAAGAACCTGATCGGAGCCCTGCTGTTCG
ACAGCGGCGAAACAGCCGAGGCCACCCGGCTGAAGAGAACCGCCAGAAGAAGATACACCAGACGGAAGAACCGGATCTGC
TATCTGCAAGAGATCTTCAGCAACGAGATGGCCAAGGTGGACGACAGCTTCTTCCACAGACTGGAAGAGTCCTTCCTGGT
GGAAGAGGATAAGAAGCACGAGCGGCACCCCATCTTCGGCAACATCGTGGACGAGGTGGCCTACCACGAGAAGTACCCCA
CCATCTACCACCTGAGAAAGAAACTGGTGGACAGCACCGACAAGGCCGACCTGCGGCTGATCTATCTGGCCCTGGCCCAC
ATGATCAAGTTCCGGGGCCACTTCCTGATCGAGGGCGACCTGAACCCCGACAACAGCGACGTGGACAAGCTGTTCATCCA
GCTGGTGCAGACCTACAACCAGCTGTTCGAGGAAAACCCCATCAACGCCAGCGGCGTGGACGCCAAGGCCATCCTGTCTG
CCAGACTGAGCAAGAGCAGACGGCTGGAAAATCTGATCGCCCAGCTGCCCGGCGAGAAGAAGAATGGCCTGTTCGGAAAC
CTGATTGCCCTGAGCCTGGGCCTGACCCCCAACTTCAAGAGCAACTTCGACCTGGCCGAGGATGCCAAACTGCAGCTGAG
CAAGGACACCTACGACGACGACCTGGACAACCTGCTGGCCCAGATCGGCGACCAGTACGCCGACCTGTTTCTGGCCGCCA
AGAACCTGTCCGACGCCATCCTGCTGAGCGACATCCTGAGAGTGAACACCGAGATCACCAAGGCCCCCCTGAGCGCCTCT
ATGATCAAGAGATACGACGAGCACCACCAGGACCTGACCCTGCTGAAAGCTCTCGTGCGGCAGCAGCTGCCTGAGAAGTA
CAAAGAGATTTTCTTCGACCAGAGCAAGAACGGCTACGCCGGCTACATTGACGGCGGAGCCAGCCAGGAAGAGTTCTACA
AGTTCATCAAGCCCATCCTGGAAAAGATGGACGGCACCGAGGAACTGCTCGTGAAGCTGAACAGAGAGGACCTGCTGCGG
AAGCAGCGGACCTTCGACAACGGCAGCATCCCCCACCAGATCCACCTGGGAGAGCTGCACGCCATTCTGCGGCGGCAGGA
AGATTTTTACCCATTCCTGAAGGACAACCGGGAAAAGATCGAGAAGATCCTGACCTTCCGCATCCCCTACTACGTGGGCC
CTCTGGCCAGGGGAAACAGCAGATTCGCCTGGATGACCAGAAAGAGCGAGGAAACCATCACCCCCTGGAACTTCGAGGAA
GTGGTGGACAAGGGCGCTTCCGCCCAGAGCTTCATCGAGCGGATGACCAACTTCGATAAGAACCTGCCCAACGAGAAGGT
GCTGCCCAAGCACAGCCTGCTGTACGAGTACTTCACCGTGTATAACGAGCTGACCAAAGTGAAATACGTGACCGAGGGAA

TGAGAAAGCCCGCCTTCCTGAGCGGCGAGCAGAAAAAGGCCATCGTGGACCTGCTGTTCAAGACCAACCGGAAAGTGACC
GTGAAGCAGCTGAAAGAGGACTACTTCAAGAAAATCGAGTGCTTCGACTCCGTGGAAATCTCCGGCGTGGAAGATCGGTT
CAACGCCTCCCTGGGCACATACCACGATCTGCTGAAAATTATCAAGGACAAGGACTTCCTGGACAATGAGGAAAACGAGG
ACATTCTGGAAGATATCGTGCTGACCCTGACACTGTTTGAGGACAGAGAGATGATCGAGGAACGGCTGAAAACCTATGCC
CACCTGTTCGACGACAAAGTGATGAAGCAGCTGAAGCGGCGGAGATACACCGGCTGGGGCAGGCTGAGCCGGAAGCTGAT
CAACGGCATCCGGGACAAGCAGTCCGGCAAGACAATCCTGGATTTCCTGAAGTCCGACGGCTTCGCCAACAGAAACTTCA
TGCAGCTGATCCACGACGACAGCCTGACCTTTAAAGAGGACATCCAGAAAGCCCAGGTGTCCGGCCAGGGCGATAGCCTG
CACGAGCACATTGCCAATCTGGCCGGCAGCCCCGCCATTAAGAAGGGCATCCTGCAGACAGTGAAGGTGGTGGACGAGCT
CGTGAAAGTGATGGGCCGGCACAAGCCCGAGAACATCGTGATCGAAATGGCCAGAGAGAACCAGACCACCCAGAAGGGAC
AGAAGAACAGCCGCGAGAGAATGAAGCGGATCGAAGAGGGCATCAAAGAGCTGGGCAGCCAGATCCTGAAAGAACACCCC
GTGGAAAACACCCAGCTGCAGAACGAGAAGCTGTACCTGTACTACCTGCAGAATGGGCGGGATATGTACGTGGACCAGGA
ACTGGACATCAACCGGCTGTCCGACTACGATGTGGACCATATCGTGCCTCAGAGCTTTCTGAAGGACGACTCCATCGACA
ACAAGGTGCTGACCAGAAGCGACAAGAACCGGGGCAAGAGCGACAACGTGCCCTCCGAAGAGGTCGTGAAGAAGATGAAG
AACTACTGGCGGCAGCTGCTGAACGCCAAGCTGATTACCCAGAGAAAGTTCGACAATCTGACCAAGGCCGAGAGAGGCGG
CCTGAGCGAACTGGATAAGGCCGGCTTCATCAAGAGACAGCTGGTGGAAACCCGGCAGATCACAAAGCACGTGGCACAGA
TCCTGGACTCCCGGATGAACACTAAGTACGACGAGAATGACAAGCTGATCCGGGAAGTGAAAGTGATCACCCTGAAGTCC
AAGCTGGTGTCCGATTTCCGGAAGGATTTCCAGTTTTACAAAGTGCGCGAGATCAACAACTACCACCACGCCCACGACGC
CTACCTGAACGCCGTCGTGGGAACCGCCCTGATCAAAAAGTACCCTAAGCTGGAAAGCGAGTTCGTGTACGGCGACTACA
AGGTGTACGACGTGCGGAAGATGATCGCCAAGAGCGAGCAGGAAATCGGCAAGGCTACCGCCAAGTACTTCTTCTACAGC
AACATCATGAACTTTTTCAAGACCGAGATTACCCTGGCCAACGGCGAGATCCGGAAGCGGCCTCTGATCGAGACAAACGG
CGAAACCGGGGAGATCGTGTGGGATAAGGGCCGGGATTTTGCCACCGTGCGGAAAGTGCTGAGCATGCCCCAAGTGAATA
TCGTGAAAAAGACCGAGGTGCAGACAGGCGGCTTCAGCAAAGAGTCTATCCTGCCCAAGAGGAACAGCGATAAGCTGATC
GCCAGAAAGAAGGACTGGGACCCTAAGAAGTACGGCGGCTTCGACAGCCCCACCGTGGCCTATTCTGTGCTGGTGGTGGC
CAAAGTGGAAAAGGGCAAGTCCAAGAAACTGAAGAGTGTGAAAGAGCTGCTGGGGATCACCATCATGGAAAGAAGCAGCT
TCGAGAAGAATCCCATCGACTTTCTGGAAGCCAAGGGCTACAAAGAAGTGAAAAAGGACCTGATCATCAAGCTGCCTAAG
TACTCCCTGTTCGAGCTGGAAAACGGCCGGAAGAGAATGCTGGCCTCTGCCGGCGAACTGCAGAAGGGAAACGAACTGGC
CCTGCCCTCCAAATATGTGAACTTCCTGTACCTGGCCAGCCACTATGAGAAGCTGAAGGGCTCCCCCGAGGATAATGAGC
AGAAACAGCTGTTTGTGGAACAGCACAAGCACTACCTGGACGAGATCATCGAGCAGATCAGCGAGTTCTCCAAGAGAGTG
ATCCTGGCCGACGCTAATCTGGACAAAGTGCTGTCCGCCTACAACAAGCACCGGGATAAGCCCATCAGAGAGCAGGCCGA
GAATATCATCCACCTGTTTACCCTGACCAATCTGGGAGCCCCTGCCGCCTTCAAGTACTTTGACACCACCATCGACCGGA
AGAGGTACACCAGCACCAAAGAGGTGCTGGACGCCACCCTGATCCACCAGAGCATCACCGGCCTGTACGAGACACGGATC
GACCTGTCTCAGCTGGGAGGCGACAAAAGGCCGGCGGCCACGAAAAAGGCCGGCCAGGCAAAAAAGAAAAAGGCTAGCGG
AAGCGGAGCCACTAACTTCTCCCTGTTGAAACAAGCAGGGGATGTCGAAGAGAATCCCGGGCCAATGACCGAGTACAAGC
CCACGGTGCGCCTCGCCACCCGCGACGACGTCCCCAGGGCCGTACGCACCCTCGCCGCCGCGTTCGCCGACTACCCCGCC
ACGCGCCACACCGTCGATCCGGACCGCCACATCGAGCGGGTCACCGAGCTGCAAGAACTCTTCCTCACGCGCGTCGGGCT
CGACATCGGCAAGGTGTGGGTCGCGGACGACGGCGCCGCGGTGGCGGTCTGGACCACGCCGGAGAGCGTCGAAGCGGGGG
CGGTGTTCGCCGAGATCGGCCCGCGCATGGCCGAGTTGAGCGGTTCCCGGCTGGCCGCGCAGCAACAGATGGAAGGCCTC
CTGGCGCCGCACCGGCCCAAGGAGCCCGCGTGGTTCCTGGCCACCGTCGGAGTCTCGCCCGACCACCAGGGCAAGGGTCT
GGGCAGCGCCGTCGTGCTCCCCGGAGTGGAGGCGGCCGAGCGCGCCGGGGTGCCCGCCTTCCTGGAGACCTCCGCGCCCC
GCAACCTCCCCTTCTACGAGCGGCTCGGCTTCACCGTCACCGCCGACGTCGAGGTGCCCGAAGGACCGCGCACCTGGTGC
ATGACCCGCAAGCCCGGTGCCTGAAATAAAAGATCTTTATTTTCATTAGATCTGTGTGTTGGTTTTTTGTGTAAGCTTTG
GTAACCACGTGCGGACCGAGCGGCCGCAGGAACCCCTAGTGATGGAGTTGGCCACTCCCTCTCTGCGCGCTCGCTCGCTC
ACTGAGGCCGGGCGACCAAAGGTCGCCCGACGCCCGGGCTTTGCCCGGGCGGCCTCAGTGAGCGAGCGAGCGCGCAGCTG
CCTGCAGGGGCGCCTGATGCGGTATTTTCTCCTTACGCATCTGTGCGGTATTTCACACCGCATACGTCAAAGCAACCATA
GTACGCGCCCTGTAGCGGCGCATTAAGCGCGGCGGGTGTGGTGGTTACGCGCAGCGTGACCGCTACACTTGCCAGCGCCT
TAGCGCCCGCTCCTTTCGCTTTCTTCCCTTCCTTTCTCGCCACGTTCGCCGGCTTTCCCCGTCAAGCTCTAAATCGGGGG
CTCCCTTTAGGGTTCCGATTTAGTGCTTTACGGCACCTCGACCCCAAAAAACTTGATTTGGGTGATGGTTCACGTAGTGG
GCCATCGCCCTGATAGACGGTTTTTCGCCCTTTGACGTTGGAGTCCACGTTCTTTAATAGTGGACTCTTGTTCCAAACTG
GAACAACACTCAACTCTATCTCGGGCTATTCTTTTGATTTATAAGGGATTTTGCCGATTTCGGTCTATTGGTTAAAAAAT
GAGCTGATTTAACAAAAATTTAACGCGAATTTTAACAAAATATTAACGTTTACAATTTTATGGTGCACTCTCAGTACAAT
CTGCTCTGATGCCGCATAGTTAAGCCAGCCCCGACACCCGCCAACACCCGCTGACGCGCCCTGACGGGCTTGTCTGCTCC
CGGCATCCGCTTACAGACAAGCTGTGACCGTCTCCGGGAGCTGCATGTGTCAGAGGTTTTCACCGTCA-3'
